# Aminoglycoside heteroresistance in *Enterobacter cloacae* is driven by the cell envelope stress response

**DOI:** 10.1128/mbio.01699-24

**Published:** 2024-10-30

**Authors:** Ana J. Choi, Daniel J. Bennison, Esha Kulkarni, Hibah Azar, Haoyu Sun, Hanqi Li, Jonathan Bradshaw, Hui Wen Yeap, Nicholas Lim, Vishwas Mishra, Anna Crespo-Puig, Ewurabena A. Mills, Frances Davies, Shiranee Sriskandan, Avinash R. Shenoy

**Affiliations:** 1 Department of Infectious Disease, Imperial College London, London, United Kingdom; 2 Centre for Bacterial Resistance Biology, Imperial College London, London, United Kingdom; 3 NIHR Health Protection Research Unit in Healthcare Associated Infections and Antimicrobial Resistance, Imperial College London, London, United Kingdom; 4 The Francis Crick Institute, London, United Kingdom; Washington University in St. Louis School of Medicine, St. Louis, Missouri, USA; Washington University in St. Louis School of Medicine, St. Louis, Missouri, USA

**Keywords:** AMR, cell envelope stress, heteroresistance, persistence, ESKAPE, aminoglycosides

## Abstract

*Enterobacter cloacae* is a Gram-negative nosocomial pathogen of the ESKAPE (*Enterococcus, Staphylococcus, Klebsiella, Acinetobacter, Pseudomonas*, and *Enterobacter* spp.) priority group with increasing multi-drug resistance via the acquisition of resistance plasmids. However, *E. cloacae* can also display forms of antibiotic refractoriness, such as heteroresistance and tolerance. Here, we report that *E. cloacae* displays transient heteroresistance to aminoglycosides, which is accompanied with the formation of small colony variants (SCVs) with increased minimum inhibitor concentration (MIC) of gentamicin and other aminoglycosides used in the clinic, but not other antibiotic classes. To explore the underlying mechanisms, we performed RNA sequencing of heteroresistant bacteria, which revealed global gene expression changes and a signature of the CpxRA cell envelope stress response. Deletion of the *cpxRA* two-component system abrogated aminoglycoside heteroresistance and SCV formation, pointing to its indispensable role in these processes. The introduction of a constitutively active allele of *cpxA* led to high aminoglycoside MICs*,* consistent with cell envelope stress response driving these behaviors in *E. cloacae*. Cell envelope stress can be caused by environmental cues, including heavy metals. Indeed, bacterial exposure to copper increased gentamicin MIC in the wild-type but not in the Δ*cpxRA* mutant. Moreover, copper exposure also elevated the gentamicin MICs of clinical isolates from bloodstream infections, suggesting that CpxRA- and copper-dependent aminoglycoside resistance is broadly conserved in *E. cloacae* strains. Altogether, we establish that *E. cloacae* relies on transcriptional reprogramming via the envelope stress response pathway for transient resistance to a major class of frontline antibiotic.

**IMPORTANCE:**

*Enterobacter cloacae* is a bacterium that belongs to the WHO high-priority group and an increasing threat worldwide due its multi-drug resistance. *E. cloacae* can also display heteroresistance, which has been linked to treatment failure. We report that *E. cloacae* shows heteroresistance to aminoglycoside antibiotics. These are important frontline microbicidal drugs used against Gram-negative bacterial infections; therefore, understanding how resistance develops among sensitive strains is important. We show that aminoglycoside resistance is driven by the activation of the cell envelope stress response and transcriptional reprogramming via the CpxRA two-component system. Furthermore, heterologous activation of envelope stress via copper, typically a heavy metal with antimicrobial actions, also increased aminoglycoside MICs of the *E. cloacae* type strain and clinical strains isolated from bloodstream infections. Our study suggests aminoglycoside recalcitrance in *E. cloacae* could be broadly conserved and cautions against the undesirable effects of copper.

## INTRODUCTION

*Enterobacter cloacae* is a Gram-negative opportunistic pathogen that can cause life-threatening hospital-acquired infections (1, 2). These include bloodstream infections and sepsis, peritonitis, and lower respiratory tract and urinary tract infections. Due to its natural and acquired resistance to several classes of antibiotic, *E. cloacae* is included in the ESKAPE (*Enterococcus, Staphylococcus, Klebsiella, Acinetobacter, Pseudomonas*, and *Enterobacter* spp.) group of priority multi-drug-resistant pathogens (1, 3). *E. cloacae* is the type species of the growing *Enterobacter cloacae* complex group of bacteria, and the third leading cause of death from drug-resistant *Enterobacteriaceae* infection after *Escherichia coli* and *Klebsiella pneumoniae* worldwide (4). *E. cloacae* often cause life-threatening nosocomial bloodstream infections in both adults and neonates (5, 6). Therefore, a better understanding of mechanisms of antimicrobial resistance in *E. cloacae* is critical for ensuring continued efficacy of current frontline drugs.

Antibiotic resistance typically evolves through acquisition of plasmids encoding antibiotic-inactivating enzymes, mutation of antibiotic targets, increased drug efflux, and limiting drug uptake (7). These mechanisms result in a stable, heritable increase in the minimum inhibitor concentration (MIC) of an antibiotic, which subsequently becomes ineffective in treatment. Less common, but equally formidable, threats to treatment are mechanisms of recalcitrance such as heteroresistance, tolerance, persistence, and small colony variants (SCVs), among others (8, 9). In heteroresistance, a subpopulation within a clonal bacterial culture spontaneously becomes antibiotic resistant (i.e., displays high MIC, typically >8× higher than that of the culture). Tolerance leads to the survival of a bacterial population for long periods at high doses of antibiotic without an increase in MIC. When a subpopulation becomes tolerant, it is also called persistence or heterotolerance (which is consistent with the “heteroresistance” terminology) (10). SCVs, i.e., bacteria that form small colonies, are also less susceptible to bactericidal antibiotics because of a slower growth rate or longer lag phase (11). Heterogeneous antibiotic susceptibility of bacteria within a culture results in a characteristic biphasic antibiotic time-to-kill curve due to the rapid killing of the majority of the population and slower killing or longer survival of the antibiotic refractory subpopulation (8). Worryingly, antibiotic heteroresistance, tolerance, and SCV formation among priority pathogens have emerged as major threats to treatment in the clinic (8, 11, 12).

Increasing antibiotic resistance is a major concern in *E. cloacae*, which is naturally resistant to most β-lactams and extended spectrum cephalosporins (13), and is fast acquiring carbapenem-resistance plasmids (14). Plasmid-acquired resistance to other antibiotics, including polymyxins and fosfomycin, is also on the rise (15, 16). Molecular mechanisms leading to heteroresistance remain poorly understood; however, unstable increase in the copy number of antibiotic-resistance genes has been reported in *Enterobacteriaceae* (8, 17). Stress-induced transcriptional changes that lead to lipopolysaccharide (LPS) modifications can contribute to colistin heteroresistance in *E. cloacae* complex species (18). Colistin heteroresistance in *E. cloacae* complex species is lineage-dependent due to natural sequence variations within *phoPQ* genes, which affect their activity and expression of genes responsible for colistin-protective LPS modifications (15). More importantly, colistin heteroresistance has been linked to treatment failure in *E. cloacae* (19). Non-genetic mechanisms of heteroresistance are also recognized, e.g., due to stochastically increased expression of efflux pumps that promote nalidixic acid resistance (20). However, mechanisms of heteroresistance against aminoglycosides are not known.

Aminoglycosides bind to the 30S ribosomal subunit and interfere with protein translation. However, unlike other drugs that target the ribosome, aminoglycosides are bactericidal, probably due to error-prone translation and protein misfolding which contribute to toxicity (21–23). As aminoglycosides are charged molecules that do not easily enter eukaryotic cells, they are often used in “gentamicin-protection assays” to kill extracellular bacteria and measure bacteria that invaded into host cells and are protected from the antibiotic (24–26). During our studies on macrophage infection by the gentamicin-sensitive type strain of *E. cloacae*, namely ATCC 13047 [originally isolated from a spinal fluid specimen (27)], we were surprised that extracellular bacteria were not eliminated by 100 mg.L^−1^ gentamicin even at 5 h post-treatment. As aminoglycosides are integral to treatment options against sepsis and multi-drug-resistant Gram-negative infections (28), we decided to investigate this unexpected behavior further. Here, we report aminoglycoside heteroresistance in *E. cloacae,* which is associated with a subpopulation that forms unstable SCVs with higher aminoglycoside MICs. We find that increased expression of the cell envelope stress response by the CpxRA two-component system is indispensable for heteroresistance. We further show that activation of CpxRA by copper also triggers aminoglycoside resistance in *E. cloacae* and aminoglycoside-sensitive clinical isolates collected from adult bloodstream infections as part of the Bioresource in Adult Infectious Diseases (BioAID) cohort study (29). Our studies highlight potential risks and provide clarity on mechanisms of phenotypic aminoglycoside resistance in *E. cloacae*.

## RESULTS

### A subpopulation in clonal *E. cloacae* cultures displays gentamicin resistance

**Fig 1:**
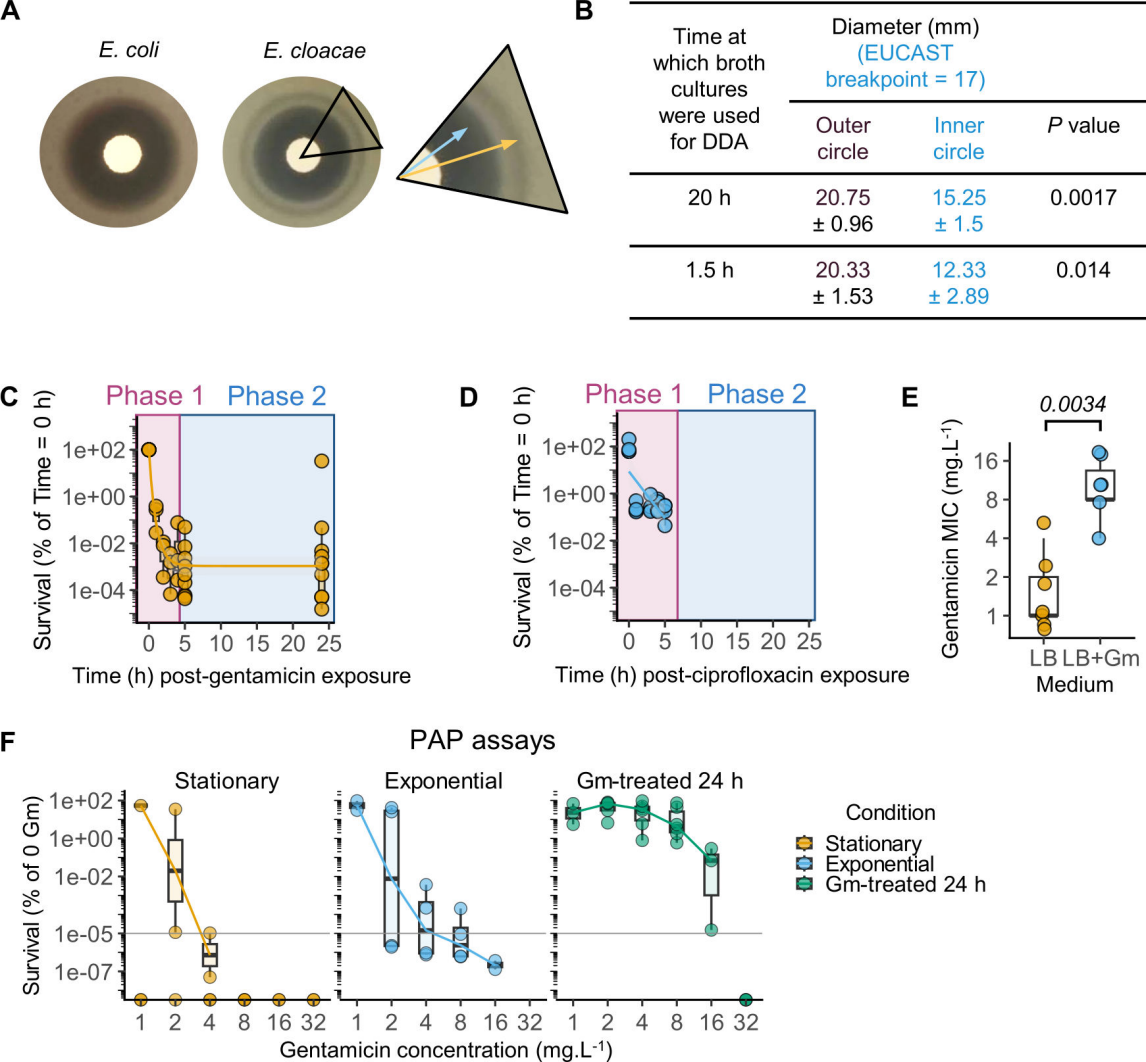
*E. cloacae* cultures contain a subpopulation that is resistant to gentamicin (Gm). (**A and B**) Representative images from disk-diffusion assays (DDA) testing gentamicin sensitivity of *E. coli* ATCC 11775 and *E. cloacae* ATCC 13047 (**A**). Inset shows *E. cloacae* “halo” inside the zone of clearance, whose diameters are shown in (**B**). Mean ± SD of independent experiments indicated; cultures were sampled at 20 h (stationary phase; *n* = 4) or 1.5 h (exponential phase; *n* = 3) for spreading on plates for disk-diffusion assays. Two-tailed *P*-values for comparison of outer and inner diameters from Student’s *t*-tests. (**C and D**) Time-to-kill curves for gentamicin (20 mg.L^−1^) (**C**) and ciprofloxacin (0.5 mg.L^−1^) (**D**) showing the percentage survival of *E. cloacae* over time. Cultures were treated with the antibiotic, samples were collected, washed, and plated on LB agar to quantify viable colony-forming units (CFU).mL^−1^. Graphs show first-order decay curve fit to data, and rapid (Phase 1) and slow (Phase 2) killing phases are marked. No viable CFUs detected after 5 h post-ciprofloxacin treatment (< 300 CFU.mL^−1^). Number of independent experiments as follows: C, *n* = 11; D, *n* = 4. (**E**) MIC measured by broth microdilution for *E. cloacae* grown in LB without or with Gm (20 mg.L^−1^) for 24 h as in experiments in C. Data from *n* = 7 (LB) and *n* = 6 (Gm) independent experiments. Two-tailed *P*-value from a Mann-Whitney *U*-test. (**F**) Population analysis profiling (PAP) assays of *E. cloacae* cultures in stationary or exponential phase or following gentamicin treatment (20 mg.L^−1^) for 24 h as labeled. Bacteria were plated on Mueller-Hinton agar plates with the indicated concentrations of gentamicin. Percentage survival relative to plates without antibiotic is plotted. Data from *n* = 4 independent experiments shown. In C–F, the box is the interquartile range (IQR), the horizontal line is the median, and the whiskers depict 1.5xIQR.

We initially observed incomplete killing of extracellular *E. cloacae* ATCC 13047 during gentamicin-protection assays in cell culture experiments, and proceeded to confirm that this strain is susceptible to gentamicin and does not grow when inoculated in lysogeny broth (LB) containing 10 mg.L^−1^–20 mg.L^−1^ gentamicin (~10×–20× MIC). However, to our surprise, in disk-diffusion assays alongside a gentamicin-susceptible reference strain of *E. coli* (ATCC 11775), we observed a “halo” of growth of *E. cloacae* within the zone of clearance (Fig. 1A and B). As expected, *E. coli* showed zones of clearance ≥17 mm in diameter, indicating susceptibility to gentamicin based on European Committee on Antimicrobial Susceptibility Testing (EUCAST) guidelines (30). The formation of an “inner” zone ≤17 mm suggested that *E. cloacae* displays a form of heterogenous resistance to gentamicin. Notably, time-dependent measurements did not show a difference in zone of clearance, as similar “halos” were observed when bacteria were sampled at 1.5 h (exponential phase) or 20 h (stationary phase) cultures for disk-diffusion assays (Fig. 1B), indicating that *E. cloacae* cultures contain a mixture of bacteria with different antibiotic susceptibilities irrespective of growth phase in broth cultures. As these observations were suggestive of a heterogenous susceptibility to gentamicin, we investigated this phenomenon further using time-to-kill and broth microdilution-based MIC assays which are more reliable in detecting the forms of antibiotic refractoriness.

Treatment of exponentially growing *E. cloacae* with gentamicin led to ~10^4^- to 10^5^-fold reduction in viable bacterial colony-forming units (CFU) between 2 and 5 h post-treatment (Fig. 1C). Analyses of data from time-to-kill assays indicated biphasic killing kinetics by gentamicin, with rapid early killing (Phase 1) and a slower late phase (Phase 2; Fig. 1C). Notably, this behavior was limited to gentamicin, as ciprofloxacin, a fluoroquinolone also used against sepsis, led to complete clearance within 5 h (Fig. 1D). Based on these experiments, we conclude that a subpopulation of *E. cloacae* ATCC 13047 can overcome gentamicin.

To assess whether the surviving bacteria had elevated MIC (i.e., were heteroresistant) or were tolerant (i.e., no change in MIC), we performed broth microdilutions of parental *E. cloacae* and those after 24-h exposure to gentamicin. The median MIC of *E. cloacae* was ~1 mg.L^−1^ (expectedly, lower than EUCAST breakpoint of 2 mg.L^−1^), which increased to ~8 mg.L^−1^ (higher than EUCAST breakpoint; Fig. 1E) for bacteria that survived exposure to gentamicin. These results indicating an increase in MIC of the surviving bacteria are consistent with the presence of heteroresistance within the population.

Next, we independently verified heteroresistance using population analysis profiling (PAP) assays. PAP assays are designed to detect whether a bacterial population contains a subpopulation at a frequency of ≥10^−5^ % that can survive antibiotic at eightfold higher concentration than the MIC (8, 31). Indeed, we observed ≥10^−5^ % bacteria could produce colonies on agar plates containing up to 8 mg.L^−1^ gentamicin, confirming heteroresistance in *E. cloacae* (Fig. 1F). Surprisingly, heteroresistance was only observed in exponentially growing cultures and not stationary phase cultures (Fig. 1F). Furthermore, in cultures exposed to 20 mg.L^−1^ gentamicin for 24 h (as in Fig. 1B), ~0.1 % of bacteria produced colonies on Mueller-Hinton Broth (MHB) agar containing 16 mg.L^−1^ gentamicin (Fig. 1F), which further points to the enrichment of the resistant subpopulation over time. Altogether, we conclude that exponentially growing cultures of the *E. cloacae* type strain contain a subpopulation with a higher gentamicin MIC.

### Formation of SCVs confers heteroresistance

**Fig 2:**
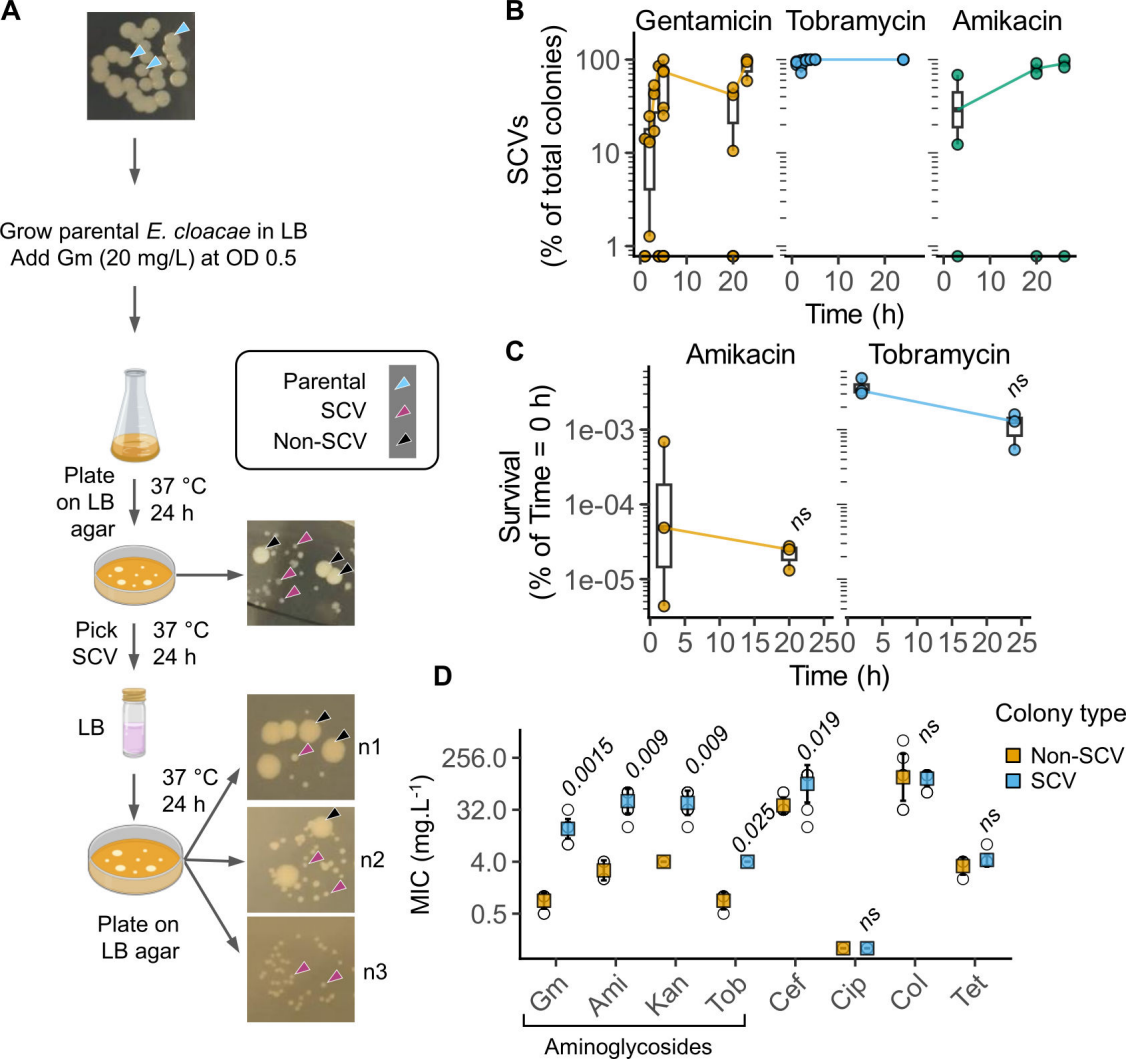
Aminoglycoside exposure of *E. cloacae* ATCC 13047 triggers SCVs with increased MICs. (**A**) Schematic depiction of the appearance of SCVs after exposure of *E. cloacae* to gentamicin (Gm) (20 mg.L^−1^), and their reversion back to “typical” colony morphotypes after growth in broth without gentamicin. Arrows used to point to colony morphotypes indicated in the legend. The images (**N1, N2, N3**) show *n* = 3 independent SCVs grown in LB for 24 h and plated on LB agar. (**B**) Percentage of SCVs after exposure to the indicated aminoglycoside for the indicated times. *E. cloacae* cultures in exponential phase were treated with gentamicin (20 mg.L^−1^), tobramycin (Tob) (20 mg.L^−1^), or amikacin (Ami) (80 mg.L^−1^) for the times as indicated. Data from *n* = 3 independent experiments. (**C**) Survival of *E. cloacae* after exposure to amikacin (80 mg.L^−1^) or tobramycin (20 mg.L^−1^) at 5 or 24 h as indicated. Data from *n* = 3 independent experiments. ns, not significant for comparison of CFU at the two time points by mixed-effects analysis of variance. (*P* > 0.05). (**D**) MICs measured by broth microdilution for non-SCVs and SCVs against Gm, Ami, kanamycin (Kan), Tob, ceftriaxone (Cef), ciprofloxacin (Cip), colistin (Col), and tetracycline (Tet). Mean (colored square) and SD shown from *n* = 8 (non-SCV) or *n* = 10 (SCV) independent experiments; open circles represent all data points. False discovery rate-adjusted two-tailed *P*-values for comparisons between SCV and non-SCV for each antibiotic from Mann-Whitney *U*-tests; ns, not significant (*P* > 0.05). In B and C, the box is the interquartile range (IQR), the horizontal line is the median, and the whiskers depict 1.5xIQR.

In gentamicin time-to-kill experiments of *E. cloacae* (Fig. 1C), we noticed the appearance of a mix of “normal” and small colonies upon plating on antibiotic-free LB agar plates (Fig. 2A and B). Importantly, after exposure to gentamicin, the proportion of SCVs increased over time (Fig. 2B), with small colonies representing ~90–100 % of the total population at 24 h post-gentamicin exposure. From here onward, we refer to the “normal-sized” colonies that have been exposed to gentamicin as non-SCVs to distinguish them from “parental” colonies formed by *E. cloacae* that has not been exposed to the antibiotic. Indeed, in PAP assays (Fig. 1F), all colonies on 8 mg.L^−1^ gentamicin LB agar plates were of the SCV morphotype. Furthermore, exposure to tobramycin and amikacin, two clinically important aminoglycosides, also resulted in *E. cloacae* SCVs whose proportion increased over time (Fig. 2B). Importantly, neither aminoglycoside could completely sterilize *E. cloacae* cultures as the number of viable bacteria did not change between 5 and 24 h post-treatment (Fig. 2C). These results indicate cultures of the *E. cloacae* type strain contain a subpopulation that can overcome aminoglycosides.

Importantly, the SCV phenotype was transient. Small colonies grown overnight in the absence of antibiotic produced both SCVs and non-SCVs, with between 0 % and 88 % of colonies of the SCV morphotype (Fig. 2A; Fig. S1A and B). To monitor the time taken by SCVs to revert, we grew four independent SCVs, non-SCVs, and parental colonies for three successive passages (1:1,000 dilution, ~10 generations per passage) without gentamicin and monitored the non-SCV frequency using spot-dilution assays over time (Fig. S1A and B). After one passage, two of the four SCV cultures had fully reverted to non-SCVs, and the remaining two had ~15 % and ~13 % reversion (24 h, Fig. S1A and B). After two passages (48 h), these cultures had, respectively, ~72 % and ~32 % non-SCVs, and after three passages (72 h), both cultures showed 100% non-SCV colony morphotypes. No SCVs were observed in cultures of parental or non-SCV colonies at any stage (Fig. S1B). These experiments indicate that individual SCVs are transient, and the rate of this reversion can vary between 24 and 72 h. Taken together, we reason that SCV formation could enable bacteria to transiently withstand aminoglycosides, and this subpopulation becomes enriched over time when exposed to aminoglycoside antibiotics and forms a larger proportion of surviving bacteria.

### SCVs have higher MICs for aminoglycosides

We hypothesized that heteroresistance arises because the subpopulation that forms SCVs has higher MIC against aminoglycosides. Indeed, MICs of SCVs against gentamicin, amikacin, kanamycin, and tobramycin were higher than that of non-SCV bacteria: ~16-fold higher for gentamicin, ~20-fold higher for amikacin, ~12-fold higher for kanamycin, and ~4-fold higher for tobramycin (Fig. 2D). These results indicate that SCVs have broad resistance to aminoglycosides with MICs higher than EUCAST breakpoints (2 mg.L^−1^). Interestingly, the MIC against ceftriaxone, a third-generation cephalosporin, also increased ~4-fold (Fig. 2D). In contrast, MICs of SCVs against other antibiotic classes, including a fluoroquinolone (ciprofloxacin), a polymyxin (colistin), and tetracycline, remained unchanged (Fig. 2D). Overall, we conclude that the SCV morphotype promotes resistance mainly against aminoglycosides.

**Fig 3:**
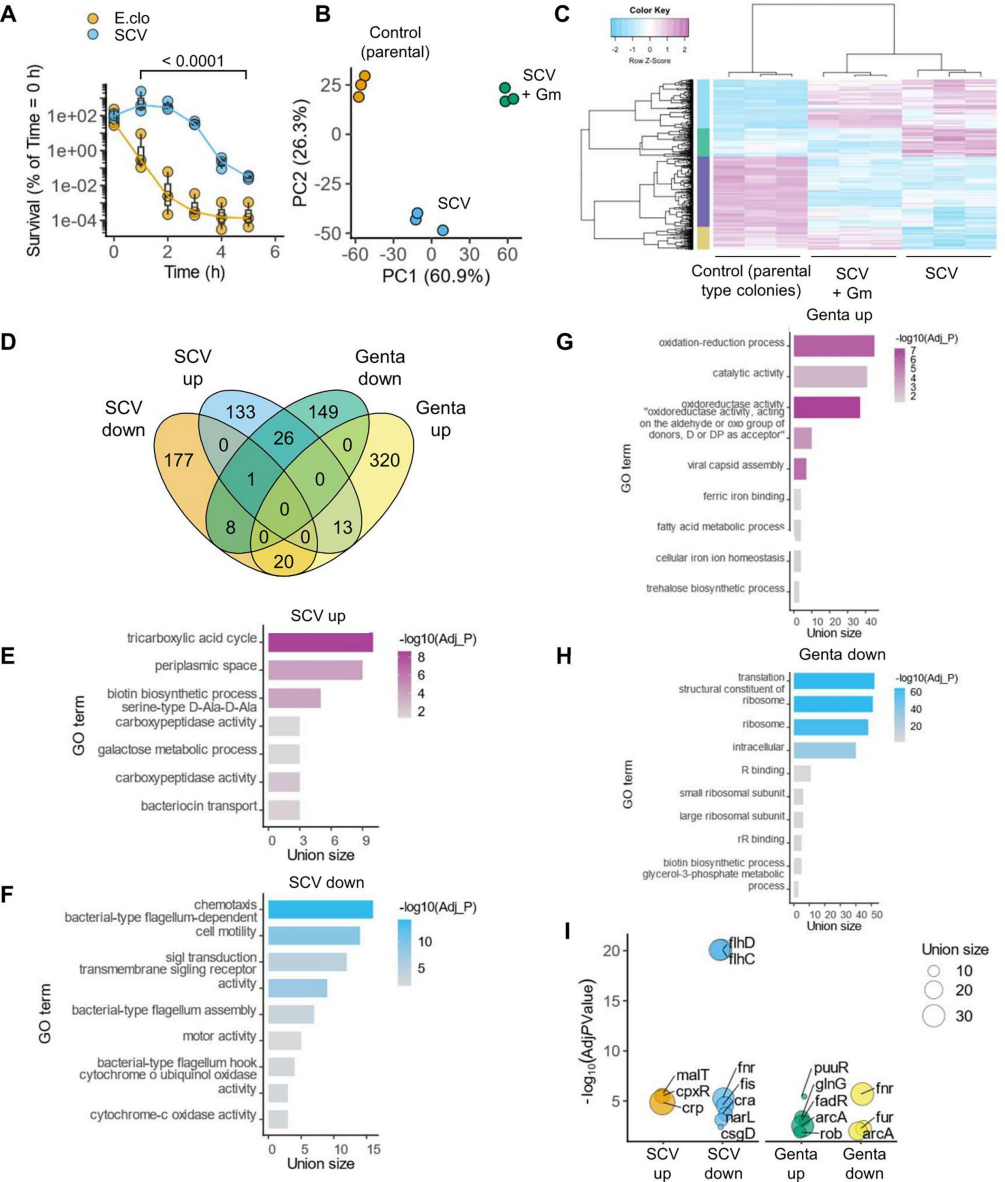
Transcriptional reprogramming drives heteroresistance to gentamicin. (**A**) Gentamicin time-to-kill curves after exposure to gentamicin (8 mg.L^−1^) for parental *E. cloacae* (E.clo) or SCV as indicated. In the graph, the box is the interquartile range (IQR), the horizontal line is the median, and the whiskers depict 1.5xIQR from *n* = 3 independent experiments. (**B and C**) Principal component analyses (**B**) and hierarchical clustering (**C**) of RNA sequencing data from the indicated growth conditions. Each dot in (**B**) represents an independent biological sample; *n* = 3 experiments. Color key in (**C**) indicates raw Z-scores of normalized log_2_ counts per million for differentially expressed genes in the indicated conditions. Gm, gentamicin (8 mg.L^−1^). (**D**) Venn diagram showing the distribution of up- and downregulated genes in the indicated conditions following RNA sequencing analyses. (**E–H**) Gene ontology (GO) terms enriched in genes up- or downregulated in the indicated comparisons. SCV, SCV vs parental; Genta, SCV given gentamicin (8 mg.L^−1^) for 2 h vs SCV without gentamicin. The union size is the number of target genes in the data set. False discovery rate (FDR)-adjusted *P*-values for GO terms plotted on -log_10_ scale. (**I**) Plot showing the statistically enriched transcription factors among differentially expressed genes as indicated. The union size is the number of target genes in the data set. Hits with FDR-adjusted *P* < 0.05 (Adj *P*-value) are plotted on a -log_10_ scale.

We next asked whether SCV morphotypes survive exposure to gentamicin for longer periods of time as compared to parental bacteria. Indeed, after exposure to 8 mg.L^−1^ gentamicin, there was no change in the viability of SCVs up to 2 h, whereas only ~10^−3^ % non-SCV survived (Fig. 3A). Similarly, ~100-fold more SCVs survived at 5 h than non-SCVs (Fig. 3A). We therefore conclude that SCVs can withstand gentamicin without loss in viability for longer periods of time as compared to the parental *E. cloacae* morphotypes.

### Global gene expression changes in *E. cloacae* exposed to gentamicin

We next sought to determine the mechanisms that promote gentamicin resistance in SCVs. Given the transient, reversible nature of the heteroresistance behavior, we hypothesized that differential gene expression empowers SCVs to resist gentamicin. To test this, we performed sequencing on mRNA from exponentially growing parental *E. cloacae* (not exposed to gentamicin) and SCVs (from 8 mg.L^−1^ LB-gentamicin plates in PAP assays). We also prepared RNA from exponentially growing SCVs treated with gentamicin (8 mg.L^−1^) for 2 h, a time point at which there is no change in viable bacterial numbers (Fig. 3A). Consistent with our hypothesis, principal component analyses of RNA sequencing data revealed clear separation of the three treatment groups, indicative of distinct gene expression programs in these conditions (Fig. 3B). Unsupervised hierarchical clustering similarly resulted in neighboring grouping of the three categories with many differentially regulated genes (Fig. 3C). Overall, at log_2_(fold-change) ≥2 or ≤ −2 cut-offs, there were 377 differentially expressed genes when comparing SCVs vs parental colonies, and 536 differentially expressed genes after treatment of SCVs with gentamicin (Fig. 3D; Tables S1 and S2). Notably, the highest upregulated genes in SCV were periplasmic proteases, chaperones, inner and outer membrane proteins, and maltose or trehalose transport genes; among the most downregulated were the flagellar apparatus and fimbrial biosynthesis genes (Tables S1 and S2).

To identify metabolic processes and signaling pathways that could be responsible for gentamicin heteroresistance, we performed enrichment analyses on up- and downregulated genes. Among the upregulated genes in SCVs were gene ontology (GO) terms representing periplasmic processes, the TCA cycle intermediates for supplying basic metabolic building blocks, biotin synthesis, galactose metabolism, and bacteriocin transport (Fig. 3E; Table S3). In contrast, processes such as chemotaxis and motility and cytochrome c oxidase activity were downregulated in SCVs (Fig. 3F; Table S3). Upon treatment with gentamicin, pathways involved in redox reactions and withstanding reactive oxygen species (ROS), iron uptake, and trehalose synthesis were enriched (Fig. 3G; Table S3). Not surprisingly, processes involving protein translation and ribosomal structure were markedly downregulated after gentamicin treatment (Fig. 3H; Table S3). Together, these analyses indicate that *E. cloacae* undergoes a broad shift in bioenergetics and substrate utilization with a switch to anaerobic respiration, reduced motility, and altered processes in the periplasm and cell wall.

We next investigated transcription factors that might drive these gene expression changes. Because few data sets are available from direct studies on *E. cloacae,* we relied on cross-species gene mapping from studies in *E. coli* MG1655 (available on BioCyc) alongside manual curation. Notably, genes with the highest fold-change were targets of *cpxR* (ECL_05064), which is activated by its cognate sensor kinase *cpxA* (ECL_05065) in response to cell envelope stress (32) (Fig. 3I). Targets of the global regulator such as *crp* (ECL_04734) and the maltose operon regulator *malT* (ECL_04784) were also upregulated, which indicated catabolite repression-like responses (Fig. 3I). Among downregulated genes were many targets of *flhD* (ECL_01400) and *flhC* (ECL_01401), which is consistent with the reduced expression of the flagellar apparatus and chemotaxis genes (Table S1). Additionally, *fnr* (ECL_02259), *csgD* (ECL_02600), *narL* (ECL_01619), *cra* (ECL_00876), and *fis* (ECL_04646) targets were enriched among downregulated genes in SCVs. Gentamicin treatment led to enrichment of *puuR* (ECL_02228), *glnG* (ECL_05113), *fadR* (ECL_01510), *rob* (ECL_00809), and *arcA* (ECL_00811) targets among upregulated genes, whereas *fnr* (ECL_02259), *fur* (ECL_03032), and *arcA* (ECL_00811) targets were downregulated (Fig. 3I). Taken together, we conclude that gentamicin exposure results in a broad activation of stress responses that promote anaerobiosis, reduced motility, and reduced cell division and growth.

### Cell envelope stress response is required for aminoglycoside heteroresistance

**Fig 4:**
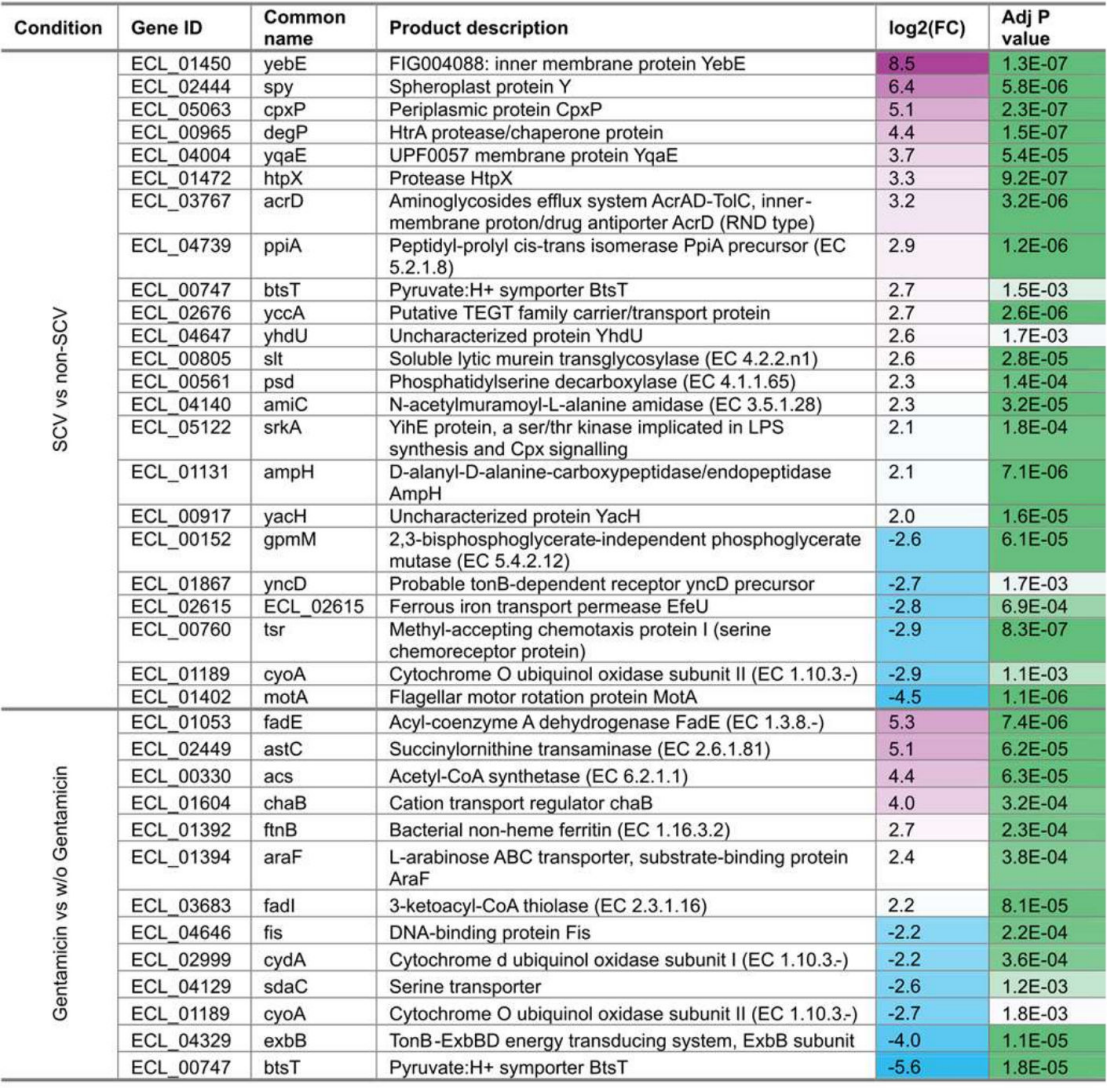
Differentially regulated genes predicted to be CpxRA targets. Differentially expressed genes in SCVs versus parental colonies and gentamicin-treated versus untreated SCV conditions as indicated. Log_2_ fold-change [log_2_(FC)] and false discovery rate-adjusted *P*-values are listed.

Several targets of the CpxRA stress response were among the most differentially regulated genes in SCV and upon further exposure to gentamicin (Fig. 4). This included the upregulation of periplasmic chaperones (*spy*, *ppiA* and *cpxP*), proteases (*degP* and *htpX*), transporters (*acrD*, *btsT*, *yccA*), and downregulation of motility and chemotaxis proteins (*motA* and *tsr*) and iron acquisition (*efeU*) (Fig. 4). Even though ROS-quenching enzymes were upregulated in SCVs, neither gentamicin MIC nor heteroresistance in PAP assays were affected by the inclusion of the ROS quencher, thiourea, in culture media (Fig. S2A and B). We conclude that ROS play no role in heteroresistance in *E. cloacae*.

**Fig 5:**
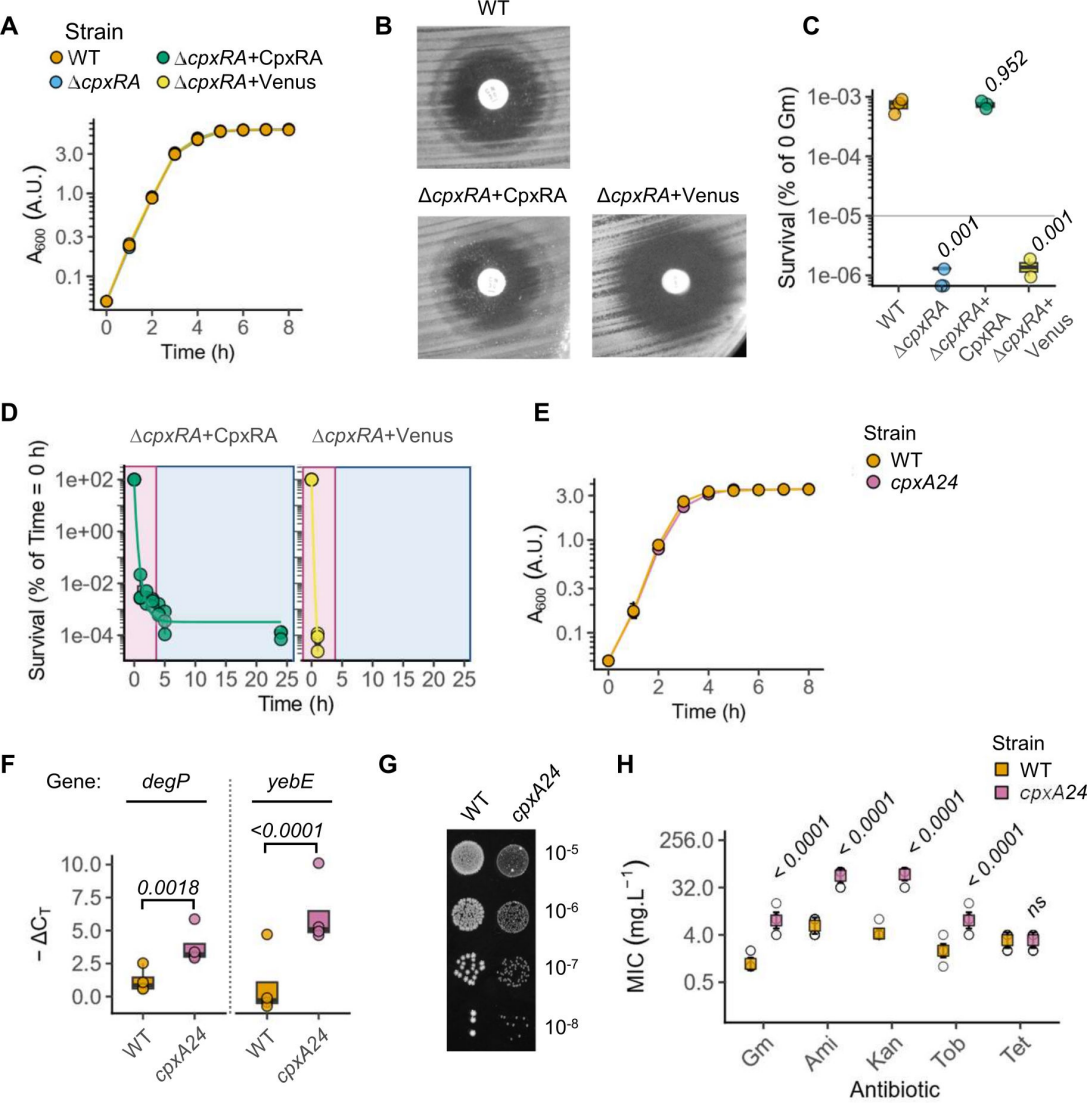
The CpxRA cell envelope stress response pathway is required for gentamicin (Gm) resistance. (**A**) Growth curves of the indicated *E. cloacae* strains plotted as mean ± SD from *n* = 3 experiments. (**B**) Representative images from gentamicin disk-diffusion assays showing *cpxRA*-dependent “halo” within the zone of clearance from *n* = 4 independent experiments. (**C**) PAP assay showing proportion of surviving bacteria of the indicated *E. cloacae* genotypes on agar plates with 8 mg.L^−1^ gentamicin. Data from *n* = 3 independent experiments. Two-tailed *P*-values for the comparisons with wild-type (WT) *E. cloacae* from mixed-effects analysis of variance (ANOVA). (**D**) Gentamicin time-to-kill curves for the indicated complemented and Δ*cpxRA* knockout *E. cloacae* showing the percentage survival over time after exposure to gentamicin (20 mg.L^−1^). Graph shows first-order decay curve fit to data from *n* = 3 independent experiments. (**E**) Growth curves of the indicated *E. cloacae* WT and *cpxA24* strains in LB plotted as mean ± SD from *n* = 3 experiments. (**F**) Quantitative Reverse Transcription Polymerase Chain Reaction (qRT-PCR) of CpxRA-target genes *degP* and *yebE* (relative to *rpoD*) in the indicated strains. Data from *n* = 4 independent experiments. (**G**) Representative images showing small colony morphology of the WT and *cpxA24* strain, plated on LB agar without antibiotics. (**H**) MICs measured by broth microdilution for the indicated strains against Gm, amikacin (Ami), kanamycin (Kan), tobramycin (Tob), and tetracycline (Tet). Mean (colored square) and SD shown from *n* = 12 independent experiments. Open circles represent all data points. False discovery rate-adjusted two-tailed *P*-values of comparisons between WT and *cpxA24* are shown from non-parametric two-way ANOVA following aligned-rank transformation. In C, D, F, and H, the box is the interquartile range (IQR), the horizontal line is the median, and the whiskers depict 1.5xIQR.

The response regulator CpxR (ECL_05064) is activated upon phosphorylation by the CpxA (ECL_05065) sensor histidine kinase that is in turn activated by altered periplasmic homeostasis and phosphorylation (32). Interestingly, CpxA may phosphorylate other response regulators, and, on the other hand, CpxR can be activated by other sensor kinases (32, 33). Therefore, to investigate the role of the CpxRA two-component system, we generated a scarless Δ*cpxRA E. cloacae* strain lacking both genes. We complemented the Δ*cpxRA* strain by introducing a single copy of *cpxRA* under their natural promoter (Δ*cpxRA* + CpxRA), and a strain containing a single copy of mVenus fluorescent protein under a constitutive promoter (34) served as a negative control (Δ*cpxRA* + Venus). All strains grew similarly in LB, which ruled out defects under standard growth conditions (Fig. 5A). We first assessed gentamicin sensitivity in disk-diffusion assays and found that the *cpxRA* knockout formed a larger zone of clearance, indicating sensitivity to gentamicin (Fig. 5B). Notably, the ring of growth (“halo”) which was seen with the wild-type *E. cloacae* was lost in the Δ*cpxRA* but was seen with the complemented strain (Fig. 1A and 5B), indicating that the unusual appearance of the zone of clearance is also *cpxRA*-dependent.

We next assessed gentamicin heteroresistance in PAP assays, which revealed that the Δ*cpxRA* strain completely lost heteroresistance and did not form SCVs (Fig. 5C), pointing to an indispensable role for cell envelope stress response in these processes. Lack of heteroresistance in Δ*cpxRA* bacteria was restored to wild-type levels in the complemented strain (Fig. 5C); the Δ*cpxRA* + Venus strain behaved similarly to the scarless Δ*cpxRA* strain, indicating that insertion of genes at the attTn7 site downstream of the *glmS* locus did not have adverse effects in *E. cloacae* (Fig. 5C). Together, these results conclusively show that the cell envelope stress response driven by the CpxRA regulon is indispensable for gentamicin heteroresistance in *E. cloacae*.

Next, we asked whether the loss of *cpxRA* also sensitized *E. cloacae* to rapid and complete killing by gentamicin. Indeed, the Δ*cpxRA +* Venus strain was rapidly killed with no detectable CFU after 1 h post-treatment (Fig. 5D); the Δ*cpxRA +* CpxRA behaved similarly to the wild-type strain displaying a biphasic kill curve (Fig. 5D), similar to that seen with the wild-type strain (Fig. 1C). Together, these results conclusively show that the cell envelope stress response driven by the CpxRA regulon is indispensable for gentamicin heteroresistance in *E. cloacae*.

In PAP assays, we observed heteroresistance during exponential phase, but not stationary phase growth (Fig. 1F). We therefore assessed CpxRA signaling by measuring the temporal change in mRNA abundance of its target, *yebE,* in wild-type and Δ*cpxRA E. cloacae* cultures. Interestingly, *yebE* mRNA abundance was comparable and higher in both strains in the stationary phase (Fig. S2C). However, in the exponential phase, *yebE* abundance was ~8-fold lower in Δ*cpxRA* as compared to wild-type bacteria (Fig. S2D). We therefore reason that the ability to turn on CpxRA activity in exponential phase may enable wild-type *E. cloacae* to overcome aminoglycosides in the exponential phase.

We next asked whether constitutively active envelope stress response can recapitulate these phenotypes. In *E. coli*, the *cpxA24* allele lacking 32 aa in the periplasmic loop of the sensor kinase CpxA is constitutively active (32). As *E. cloacae* CpxA (ECL_05065) is >95% identical in amino acid sequence to the *E. coli* CpxA (Fig. S3), we generated a similar deletion and introduced this allele (*cpxA24*) at the native genomic locus in *E. cloacae*. Wild-type and *cpxA24 E. cloacae* grew similarly in normal growth conditions, ruling out broad defects in growth rates in standard media (Fig. 5E). Quantitative Reverse Transcription Polymerase Chain Reaction (qRT-PCR) of two CpxRA-target genes that were upregulated in SCVs, *degP* and *yebE* (Fig. 4), confirmed their elevated expression in the *cpxA24* strain (Fig. 5F)*,* verifying that the envelope stress response is constitutively active in this strain. Interestingly, *cpxA24* formed SCVs even without aminoglycoside exposure (Fig. 5G), pointing to its contribution to the small colony morphotype.

Notably, *E. cloacae cpxA24* also had higher MICs for aminoglycosides, but not tetracycline (Fig. 5H), which is similar to that seen with SCVs from PAP assays (Fig. 2D). Together with the results on SCVs, these findings in *E. cloacae cpxA24* establish a pivotal role for the cell envelope stress response in aminoglycoside resistance.

### Copper- and CpxRA-driven aminoglycoside resistance in *E. cloacae* strains

**Fig 6:**
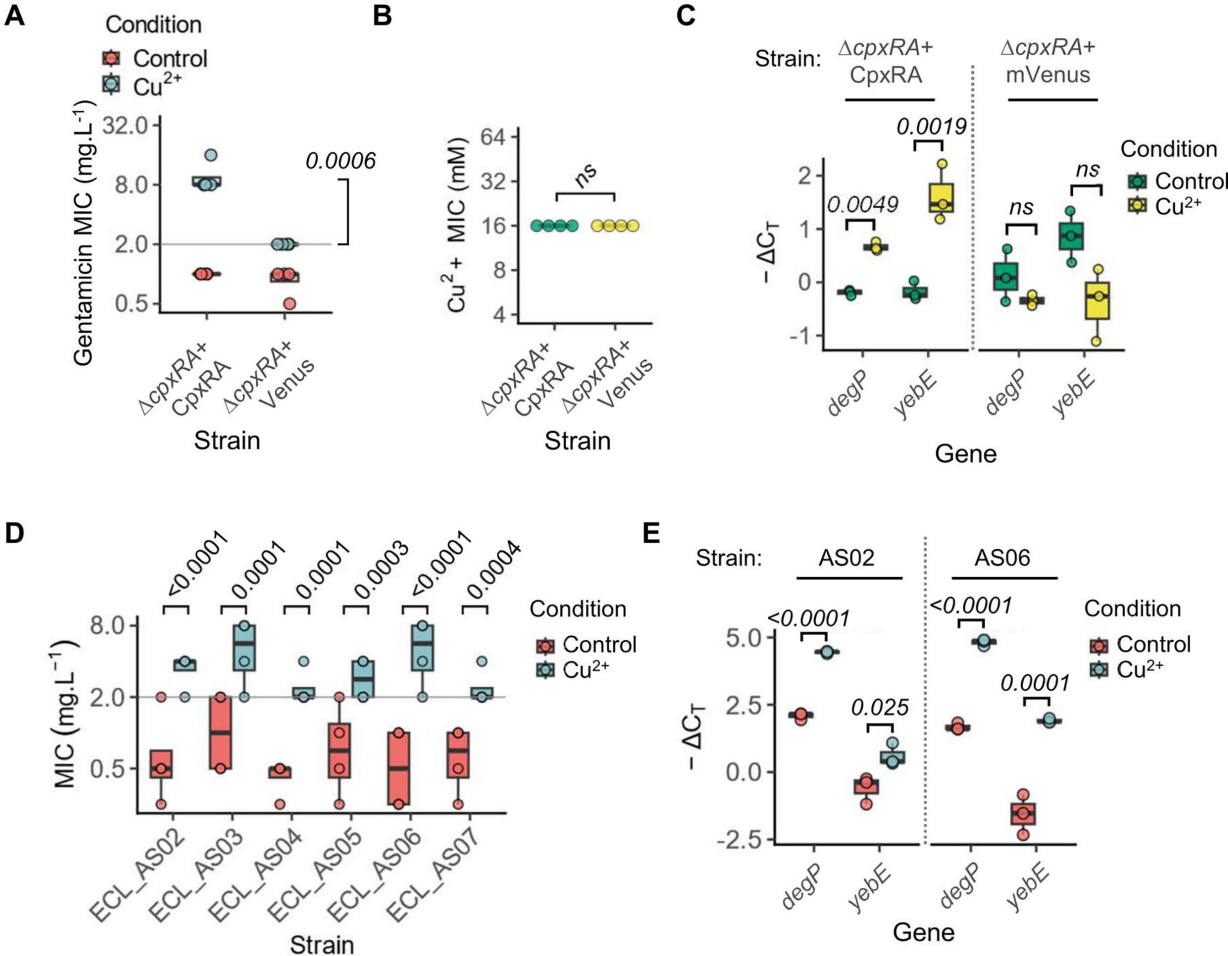
Exposure to copper increases gentamicin MIC via *cpxRA*. (**A**) Gentamicin MIC measured by broth microdilution for the indicated complemented or Δ*cpxRA* knockout *E. cloacae*. MICs were measured in the absence or presence of 4 mM copper sulfate. Data from *n* = 4 independent experiments. False discovery rate (FDR)-adjusted two-tailed *P*-value for comparison between the MIC of the two genotypes in the presence of Cu^2+^ from non-parametric analysis of variance (ANOVA) following aligned-rank transformation. (**B**) Cu^2+^ MIC measured by broth microdilution for the indicated complemented or Δ*cpxRA* knockout *E. cloacae*. Data from *n* = 4 independent experiments. ns, not significant, i.e., two-tailed *P*-value >0.05 for the indicated comparisons from Mann-Whitney *U*-test. (**C**) qRT-PCRs showing the fold-change in expression for the Cpx-target genes *degP* and *yebE* in the indicated strains treated with or without Cu^+2^ (4 mM) for 3 h, relative to the housekeeping reference *rho*. Data from *n* = 4 independent experiments. Two-tailed *P*-values from mixed-effects ANOVAs. (**D**) Gentamicin MICs for the indicated clinical *E. cloacae* isolates in the absence (“Control”) or presence of Cu^2+^ (4 mM copper sulfate). Data from *n* = 4 independent experiments. Gray line indicates EUCAST breakpoint. FDR-adjusted two-tailed *P*-values are shown for indicated comparisons in the presence of Cu^2+^ for each strain from non-parametric ANOVA following aligned-rank transformation. (**E**) qRT-PCR showing the expression of Cpx-target genes *degP* and *yebE* (relative to *rho*) in the indicated bloodstream isolates grown without and with Cu^+2^ (4 mM) for 3 h. Data from *n* = 4 independent experiments. Two-tailed *P*-values from mixed-effects ANOVAs. In A–E, the box is the interquartile range (IQR), the horizontal line is the median, and the whiskers depict 1.5xIQR.

We reasoned that as SCVs have high expression of the envelope stress regulon, heterologous activation of this pathway would also result in higher gentamicin resistance. The CpxRA pathway is activated in response to envelope stress by environmental factors, including Cu^2+^, which is an antimicrobial heavy metal (32). Consistent with this, *E. cloacae* exposed to 4 mM Cu^2+^ (copper sulfate) had ~6- to 16-fold higher gentamicin MIC (Fig. 6A), whereas the MIC of the Δ*cpxRA* strain did not increase above the breakpoint (2 mg.L^−1^) (Fig. 6A). Notably, both strains had a similar Cu^2+^ MIC of 16 mM, indicating that *cpxRA* are dispensable for copper resistance in *E. cloacae* (Fig. 6B). A similar increase in gentamicin MIC was seen with copper chloride (Fig. S2E), whereas magnesium sulfate, as a negative control, had no effect (Fig. S2F), emphasizing the role of copper, but not other cations, in *cpxRA-*dependent increase in gentamicin MIC. qRT-PCR revealed high expression of *degP* and *yebE* after Cu^2+^ treatment, which verified cell envelope stress activation in these conditions (Fig. 6C). Taken together, we establish that copper exposure confers aminoglycoside resistance in *E. cloacae* in a *cpxRA*-dependent manner.

We also tested strains isolated from bloodstream infections to rule out the possibility that these behaviors were limited to a laboratory strain of *E. cloacae*. We obtained seven strains isolated from patients in London as part of BioAID between 2015 and 2019 (Table 1); as ECL_AS01 was gentamicin-resistant, we did not use this strain in experiments. We measured the gentamicin MICs of all six gentamicin-sensitive clinical strains in the absence or presence of 4 mM Cu^2+^. Notably, similar to the *E. cloacae* ATCC 13047 type strain, copper sulfate and copper chloride, but not magnesium sulfate, increased gentamicin MICs for all six bloodstream isolates to levels above the EUCAST breakpoint (Fig. 6D; Fig. S2G). To verify copper-induced envelope stress, we tested the expression of envelope stress targets *degP* and *yebE* in two representative bloodstream isolates AS02 and AS06 by qRT-PCR, which revealed their elevated expression upon copper treatment (Fig. 6E). Taken together, we conclude that copper- and envelope stress-dependent enhancement of aminoglycoside resistance is not just a trait of the laboratory strain but also seen in sepsis-related isolates from patients. Altogether, our results establish that *E. cloacae* strains display aminoglycoside recalcitrance via cell envelope stress, for example, through exposure to copper.

**TABLE 1:** Details and antibiotic susceptibility of clinical strains from the BioAID cohort^*a*^^,*b*^

Strain ID	ECL_AS01	ECL_AS02	ECL_AS03	ECL_AS04	ECL_AS05	ECL_AS06	ECL_AS07
Pathogen isolated	*E. cloacae*	*E. cloacae*	*E. cloacae*	*E. cloacae*	*E. cloacae*	*E. cloacae*	*E. cloacae*
Date collected	04/2015	07/2015	05/2016	03/2017	03/2017	11/2017	09/2019
ESBL markers	Y	ND	N	ND	ND	N	ND
AmpC markers	ND	ND	Y	ND	ND	Y	ND
Gentamicin	**R**	S	S	S	S	S	S
Amikacin	S	S	S	S	S	S	S
Tobramycin	**R**	S	S	S	S	S	ND
Amoxycillin	**R**	**R**	**R**	**R**	**R**	**R**	**R**
Augmentin (Co-amoxiclav)	**R**	**R**	**R**	**R**	**R**	**R**	**R**
Aztreonam	**R**	S	S	S	S	S	S
Cefotaxime/Ceftriaxone	**R**	S	S	S	S	S	S
Cefoxitin	**R**	S	**R**	S	**R**	**R**	**R**
Ceftazadime	**R**	S	S	S	S	S	S
Ceftazidime-avibactam	ND	ND	ND	ND	ND	ND	S
Cefuroxime	**R**	S	S	S	S	S	S
Ciprofloxacin	**R**	S	S	S	S	S	S
Colistin	S	S	S	ND	ND	S	ND
Cotrimoxazole	ND	ND	ND	ND	ND	ND	S
Ertapenem	S	S	S	S	S	S	S
Meropenem	S	S	S	S	S	S	S
Tazocin (piperacillin tazobactam)	**R**	S	S	S	S	S	S
Temocillin	S	S	S	S	S	S	S
Trimethoprim	**R**	S	S	S	S	S	ND
Tigecycline	S	S	S	S	S	S	ND

^*a*^ S, sensitive; R, resistant; ND, not determined.

^*b*^ Bold text used to highlight where resistance was observed.

## DISCUSSION

Here, we established that *E. cloacae* displays heteroresistance through the spontaneous emergence of a subpopulation with high aminoglycoside MICs, which forms SCVs and has cell envelope stress response turned on. Furthermore, in *E. cloacae* and bloodstream isolates that we tested, activation of the CpxRA stress regulon with copper increased aminoglycoside MICs, pointing to its broad conservation in this important group of environmental and hospital-associated bacteria (35, 36). CpxRA has been implicated in fosfomycin resistance in *E. coli* (37, 38) and colistin resistance in *Salmonella enterica* serovar Typhimurium (39). Mutations in *cpxRA* have been linked to antibiotic resistance in clinical isolates of important Gram-negative bacteria, including *K. pneumoniae* (40), *Klebsiella aerogenes, Salmonella enterica* serovar Typhi (41), and *Proteus mirabilis* (42). In *Acinetobacter baumannii,* aminoglycoside heteroresistance results via the amplification of aminoglycoside-resistance genes (43). However, here, we report that differential gene expression via CpxRA can also confer heteroresistance in sensitive bacteria. These findings add to the interest in developing CpxA kinase inhibitors for combination therapy along with conventional antibiotics (44).

The sensor kinase CpxA phosphorylates the response regulator CpxR, which was originally identified as a conjugative pilus expression regulator (32). CpxR regulates ~90 genes in *E. coli*, many of which encode proteases (e.g., *degP*), chaperones (e.g., *dsbB*, *ppiA*), redox enzymes, transporters, and cell wall-modifying enzymes, which together rectify envelope stress (32). CpxRA can suppress chemotaxis and motility and increase adherence and drug efflux, which our RNA sequencing shows is also conserved in *E. cloacae*. Notably, SCVs were only observed after gentamicin exposure in the exponential phase even though the expression of the representative CpxRA-target gene *yebE* was higher during stationary phase in both wild-type and Δ*cpxRA* strains. Because *yebE* abundance in the stationary phase is *cpxRA*-independent, we reason that alternative transcription factors drive CpxRA-target genes in the stationary phase, e.g., *rpoS*, which upregulates envelope stress response upon entry into stationary phase (32, 45, 46). However, *yebE* expression in exponential phase growth was strictly *cpxRA-*dependent. We therefore hypothesize that basal CpxRA activity in exponential phase is required for SCV formation to overcome gentamicin exposure by rapidly turning on the envelope stress regulon. However, a limitation of these experiments is that they reflect CpxRA activity of the bulk population whereas antibiotic exposure results in SCVs formation by a small subpopulation. Future experiments using single-cell assays should clarify the cell-to-cell differences in gene expression that contribute to aminoglycoside recalcitrance via envelope stress signaling.

Transcriptional reprogramming in SCVs leads to a shift in metabolism to anaerobic respiration and increases catabolic activity to reduce the proton-motive force, which is required for aminoglycoside uptake (23). Anaerobiosis was also evident from reduced expression of electron transport chain and upregulation of predicted targets of fumarate nitrate reductase regulator (FNR) and nitrate/nitrite response regulator (NarL) (47), whereas starvation- and catabolite repression-like responses are suggested from the signature of cyclic-AMP response protein (Crp)-regulated genes (48). The upregulation of key efflux pumps (e.g., AcrAD) could facilitate gentamicin efflux (49) and contribute to heteroresistance. However, the AcrAD system is also regulated by the EvgAS and BaeSR regulons (33); therefore, it is unlikely to be the sole factor explaining CpxRA-dependent heteroresistance. Enrichment of various membrane proteins and the upregulation of maltose and trehalose transporters is suggestive of an imbalance in osmolytes in the face of envelope stress. Enzymes that counteract damage from ROS were also upregulated upon gentamicin exposure. However, ROS quenching did not alter bacterial susceptibility to gentamicin or heteroresistance, ruling out a role for ROS in aminoglycoside sensitivity in *E. cloacae*. We conclude that the CpxRA pathway drives a protective response that reduces growth and aerobic respiration and promotes protein re-folding and/or degradation.

Typically, SCVs are observed in response to auxotrophy (11), e.g., to menadione, thiamine, or heme, or mutations in the electron transport chain in *Staphylococcus aureus* (50). The slow growth rate of SCVs confers stable resistance to many classes of antibiotic, including aminoglycosides, trimethoprim-sulfamethoxazole, tetracycline, ciprofloxacin, oxacillin, clindamycin, and daptomycin (50). SCVs are also known in Gram-negative bacteria, e.g., *Salmonella enterica, E. coli, Shigella, Pseudomonas, Klebsiella, Neisseria, Brucella, Serratia, E. cloacae* (11, 51–53). In contrast, here, we describe transient small colony and high aminoglycoside-resistance phenotypes that readily revert to “typical” *E. cloacae* behaviors. To the best of our knowledge, this is the first report linking CpxRA stress response to the small colony morphotype.

Copper is well-known for its antimicrobial effects and triggers the CpxRA stress response through ROS-induced protein misfolding or mislocalization of outer membrane porins (32, 54). There is much interest in developing copper-coated surfaces and nanoparticles and copper-based antimicrobials (54, 55). Many opportunistic pathogens, including *E. cloacae*, often carry plasmids that encode genes for heavy-metal resistance, for instance, through co-selection in the presence of heavy metals and antibiotics in effluents in their environment (56). Multiple homologs of the copper sensing histidine kinase CusS are present in the *E. cloacae* genome. The ATCC 13047 strain additionally carries copper-resistance genes on the pECL_A plasmid (27), which likely explains the similar sensitivity to copper of the wild-type and Δ*cpxRA E. cloacae*.

Importantly, serum concentrations of gentamicin peak at 20 mg.L^−1^ and reduce to <8 mg.L^−1^ after ~5 h post-administration (57). Our results indicate that these concentrations would fail to eliminate the *E. cloacae* type strain or bloodstream isolates (Fig. 1D and 2B). Furthermore, copper increased gentamicin resistance to >8 mg.L^−1^ in *E. cloacae,* which is also worrying because gentamicin treatment regimens can be ineffective against bacteria with an MIC >2 mg.L^−1^ (58). Likewise, peak amikacin and tobramycin plasma concentrations range from 10 to 60 mg.L^−1^ and <10 mg.L^−1^, respectively (59–61), neither of which is sufficient to sterilize *E. cloacae in vitro*. The increase in ceftriaxone MIC in SCV is explained by the upregulation of the β-lactamase *ampC* (Fig. 3D; Tables S1 and S2), presumably due to cell envelope stress. Increased cross-resistance to ceftriaxone is also a concerning observation given the use of cephalosporins and aminoglycosides in combination therapy. Our results suggest that *E. cloacae* is cleared by ciprofloxacin *in vitro*, pointing to better efficacy against this pathogen.

A strength of our work is the use of diverse clinical bloodstream isolates from an ongoing cohort study of patients admitted to the hospital from the community with suspected sepsis (29). These isolates are from epidemiologically unrelated patients and are not biased by specific characteristics often required for archiving, such as antimicrobial susceptibility or any association with outbreaks (29). Gentamicin, amikacin, and tobramycin are the most commonly used aminoglycosides in the clinic, including against multi-drug-resistant Gram-negative infections and are more efficacious and better tolerated than colistin and tigecycline (62). Aminoglycosides are generally administered intravenously in hospital settings, although topical or oral neomycin is also used against skin infection (63, 64). Aminoglycosides do have downsides, such as nephrotoxicity and ototoxicity; however, these can be managed through appropriate dosing (63, 64). The newest aminoglycoside, plazomicin, was approved in 2018, pointing toward the continued interest in this class of broad-spectrum bactericidal antibiotics (65). Therefore, guarding against the development of aminoglycoside refractoriness in priority pathogens is critical.

Why is envelope stress activated in only a subset of *E. cloacae*? This could be due to several reasons, such as stochastic noise in CpxRA signaling which results in a subpopulation that is resistant. Alternatively, it is possible that lower gentamicin is taken up by a small proportion of bacteria that have a low proton-motive force, leading to sub-lethal effects on the translational machinery, allowing bacteria time to turn on the envelope stress. A limitation of our work is that we did not investigate whether CpxRA activation leading to heteroresistance in SCVs could be due to genomic changes (e.g., point mutations, in-dels, phase variation), or whether it arises “purely” from transcriptional noise in the CpxRA regulon. For instance, reversible mutations and SCVs within a homopolymeric tract in the *glpK* gene can promote drug tolerance in *Mycobacterium tuberculosis* strains (66, 67). Increased resistance-gene copy number confers heteroresistance in *E. coli, Salmonella,* and *Klebsiella* (17, 68). Whole genome sequencing of a large set of diverse aminoglycoside-sensitive *E. cloacae* strains could clarify whether heteroresistance arises via such genomic processes. Notably, however, despite relatedness to *Enterobacteriaceae*, *E. cloacae* demonstrates distinct regulation of the PhoPQ two-component system which underlies strain-dependent colistin heteroresistance in *E. cloacae* (18). We speculate that CpxRA signaling in members of the *E. cloacae* complex may also be unique and deserves to be investigated in-depth in the future. As the CpxRA operon can be triggered by heavy metals (e.g., copper), our results also suggest caution against the use of heavy metals as antimicrobial surface coatings to avoid inadvertent aminoglycoside heteroresistance.

## MATERIALS AND METHODS

### Source of strains and antibiotics

All strains used in this study are listed in Tables 1 and 2. Antibiotics are listed in Table 3.

**TABLE 2:** Bacterial strains used in this study

Bacterial strains	Source
*E. cloacae* subsp. *cloacae* ATCC 13047	
Type strain (wild type)	Purchased from ATCC. Originally isolated from spinal fluid.
Δ*cpxRA*	Scarless deletion mutant of ATCC 13047 lacking *cpxRA* (this study)
Δ*cpxRA* + mVenus	Δ*cpxRA* strain with a single copy of mVenus under the Ultra promoter at the attTn7 downstream of *glmS* (this study)
Δ*cpxRA* + CpxRA	Δ*cpxRA* strain with a single copy of *cpxRA* under its natural promoter at the attTn7 downstream of *glmS* (this study)
*cpxA24*	*E. cloacae* strain with a scarless chromosomal deletion of residues 93–124 of the *cpxA* gene (this study)
*E. cloacae* clinical strains	
ECL_AS01	See Table 1 for details [this study and part of BioAID collection (29)]
ECL_AS02	
ECL_AS03	
ECL_AS04	
ECL_AS05	
ECL_AS06	
*E. coli* strains	
*E. coli* ATCC 11775	Purchased from ATCC
*E. coli* MG 1655	Alex McCarthy, Imperial College London
*E. coli* MFD pir	Jean Marc Ghigho, Institute Pasteur
*E. coli* Stbl2	Invitrogen

**TABLE 3:** Sources of antibiotics used in this study

Product name	Product code, Company
Ampicillin sodium salt	10419313, Fisher Scientific
Amikacin sulfate salt	361958, Fluorochem
Ceftriaxone sodium hemiheptahydrate	C2226, Tokyo Chemical Industry
Ciprofloxacin	17850, Sigma-Aldrich
Colistin sulfate	C5647, LKT Laboratories
Gentamicin solution	G1272, Sigma-Aldrich
Kanamycin sulfate from *Streptomyces kanamyceticus*	K4000-25G, Sigma-Aldrich
Streptomycin sulfate salt	S9137-25G, Sigma-Aldrich
Tetracycline	87128, Sigma-Aldrich
Tobramycin	BS-9878A-1G, Molecular Dimensions

### *E. cloacae* strains and preparation of starting cultures for all experiments

*E. cloacae* ATCC 13047 and *E. coli* ATCC 11775 were obtained from ATCC (Table 2), and clinical isolates were collected under the BioAID study (29) (Table 1). Starter cultures (i.e., 5 mL overnight cultures) for experiments were prepared from frozen Dimethyl Sulfoxide (DMSO) stocks as recommended for studies on *E. coli* persisters to reduce variability across experiments and ensure inoculation of similar bacterial numbers into experimental culture flasks (69). Briefly, DMSO stocks were prepared as follows: a single *E. cloacae* colony (type strains or clinical isolates) from a freshly streaked out LB agar plate was inoculated into 5 mL filter-sterilized LB (L3022, Sigma-Aldrich) with ampicillin (100 mg.L^−1^) and incubated at 37 °C with shaking (180 rpm). This culture was diluted 1:100 into fresh 50 mL LB prepared in a 250 mL flask and grown at 37 °C with shaking to an optical density at 600 nm (OD_600_) of 0.5, following which 9.2 mL culture was mixed with 0.8 mL DMSO, and 100 µL aliquots were prepared in sterile screw-capped tubes and stored at −80°C. Overnight cultures for all experiments were prepared by inoculating 50 µL from DMSO stocks into 5 mL LB (approx. 2.5 × 10^5^ CFU.mL^−1^) to have a uniform number of starting bacteria in the pre-inoculum. OD_600_ of 0.1 was found to correspond to ~10^8^ CFU.mL^−1^ for *E. cloacae* by plating.

### Measurement of bacterial antibiotic susceptibility

MICs were determined by the broth microdilution method (42). MHB (70192, Sigma-Aldrich, Spain) was autoclaved and cation-adjusted (with filter-sterilized 1,000× stock of MgCl_2_ and CaCl_2_) to a final concentration of 10 mg.L^−1^ Mg^2+^ and 20 mg.L^−1^ Ca^2+^. Stationary phase cultures (37 °C, 18 h–24 h, with shaking) were washed twice with sterile phosphate buffered saline (PBS; D8537, Sigma-Aldrich) and diluted to 5 × 10^5^ CFU.mL^−1^ for experiments. Twofold dilutions of antibiotics were used (technical duplicates) in round-bottom 96-well plates, including wells without antibiotics and wells without bacteria as controls. Plates were incubated in a humidified 37 °C chamber for 24 h before noting the lowest antibiotic concentration that inhibited visible bacterial growth. MIC breakpoints from EUCAST were used to classify bacteria as susceptible or resistant to each antibiotic tested, whose sources are listed in Table S3. These were 0.5 mg.L^−1^ for ciprofloxacin, 2 mg.L^−1^ for gentamicin, tobramycin, ceftriaxone, and colistin; 4 mg.L^−1^ for tetracycline; and 8 mg.L^−1^ for amikacin and kanamycin.

For the disk-diffusion method, 100 µL of 10^8^ CFU.mL^−1^ bacterial culture was spread on MH agar plates and disks containing 10 µg gentamicin were placed on the agar surface within 15 min. Disks were prepared based on EUCAST guidelines. Plates were incubated overnight at 37 °C and the diameter of the zone of inhibition was measured and compared to the EUCAST zone diameter breakpoint (17 mm for gentamicin for *Enterobacterales*) (30).

### Antibiotic time-to-kill experiments

Mid-exponential phase (OD_600_ = 0.5) cultures (prepared from overnight cultures started with frozen stocks as above) were treated with 20 mg.L^−1^ gentamicin, 80 mg.L^−1^ amikacin, 20 mg.L^−1^ tobramycin, or 0.5 mg.L^−1^ ciprofloxacin and samples were collected at various time points, and washed twice in sterile PBS before preparing serial dilutions for enumerating viable bacterial counts. Typically, 3-, 5-, or 10-fold serial dilutions were prepared, of which 10 µL was spotted onto agar plates (two technical replicates), and spots with 3–50 colonies counted and a mean CFU.mL^−1^ calculated per experiment (detection limit: 3 × 10^2^ CFU.mL^−1^).

### PAP assays

PAP assays were performed to detect heteroresistance as described before (12, 31). Bacteria were grown overnight from DMSO stocks, inoculated at 1:100 dilution in fresh 50 mL LB in a 250 mL flask and at OD = 0.5, 10–50 mL culture was centrifuged (5,000 × *g*, 5 min), washed once in sterile PBS, and serial 3-, 5-, or 10-fold dilutions as appropriate were prepared in PBS and duplicate 10 µL spotted on agar plates containing 0, 2, 4, 8, or 16 mg.L^−1^ gentamicin. Plates were incubated overnight at 37 °C and the proportion of surviving bacteria at each concentration of gentamicin relative to gentamicin-free plate was calculated. As the gentamicin MIC for *E. cloacae* is ~1 mg.L^−1^, heteroresistance was defined as the appearance of viable bacteria at a frequency of ≥10^−7^ on plates containing 8 mg.L^−1^ gentamicin. The CFU detection limit was 10^2^ CFU.mL^−1^, and experiments used ≥10^10^ bacteria to have sufficient sensitivity to detect heteroresistance. For bacterial cultures treated with gentamicin for 24 h, which contain very low CFU.mL^−1^, 100 mL culture was centrifuged, washed twice, and used for PAP assays.

### Molecular cloning and plasmids

During our studies, we found that *E. cloacae* carrying the J23100-mRFP-331Bb [Addgene plasmid #78271, gift from Tom Ellis (70)], which confers chloramphenicol resistance and drives monomeric Red Fluorescent Protein (mRFP) expression using a strong, synthetic promoter J23100 and ribosome binding site (RBS), were poorly fluorescent. Similar lack of expression was observed with the pA1 promoter in another plasmid (not shown). The “Ultra” promoter from the pUltra series of plasmids [that confer kanamycin resistance (34)] was used to generate pJC-Ultra plasmid expressing mTurquoise2 [from pLifeAct-mTurquoise2; Addgene plasmid #36201, gift from Dorus Gadella (71)] in the 331Bb backbone, which led to detectable fluorescence. However, for reasons not yet clear, exposure to gentamicin and small colony formation led to plasmid loss. We therefore developed a plasmid that integrates at the T7 insertion site 3´ of *glmS* through homologous recombination. The conjugative pTOX6 vector (72) (R6Kγ origin of replication) was modified for this purpose by removing the rhamnose regulator (digested with blunt-end cutters StuI and BstZ17I enzymes and re-ligated) and then the Tse2 toxin (digested with FspI and HincII enzymes and re-ligated) to generate pTraCam (which retains *tra* genes for conjugative transfer and Cam^R^). Cloning of the Ultra-promoter-RBS-mTrq2 into PstI cut pTraCam via sequence and ligase independent cloning [SLIC; (73)] generated the pTraCam-Ultra-mTrq2 plasmid. A region (~500 bp) flanking the T7 insertion 3´ of *glmS* was cloned into the XhoI site of pTraCam-Ultra-mTrq2 to generate pTraCam-Ultra-mTrq2-EcloGlmS. All cloning steps were carried out in MFDpir *E. coli* (74), which was also used to conjugate this plasmid into *E. cloacae*. Conjugants were confirmed as chloramphenicol resistant and the presence of natural pECL_A and pECL_B plasmids was confirmed by PCR. Proof-reading polymerases such as Phusion or KOD were used for PCR and cloned fragments were verified by Sanger sequencing (Azenta). All primers used in this study are listed in Table 4.

**TABLE 4:** Primers used in this study

	Primer name	Primer sequence
*cpxRA* knockout	cpxA-Tox3-Fwd	GTTCTGGTCGCCCATGATGGCTGCGGTTAACGCTGTG
	cpxA-dnTox3-XhoI-Rvs	CGGCCGCGGTCCGATCGAGCTCGAGAAGGGCTGACGGAGAAGAACG
	cpxA-upTox3-XhoI-Fwd	TAAATGCATCCCGGGACGTCTCGAGTTCTGCCTGAGCACGTACAGC
	cpxA-Tox3-Rvs	ACAGCGTTAACCGCAGCCATCATGGGCGACCAGAACGTT
*cpxA24*(Δ93–124)	CpxA24_up_F	TAAATGCATCCCGGGACGTCTCGAGACCGGTACCGAGTTCACC
	CpxA24_down_R	CGGCCGCGGTCCGATCGAGCTCGAGAGATCGTTGATCATGCTGTCCAG
	CpxA24_Fwd	AGCGAAGGCCGGGTTGAGATGGTGGGC
	CpxA24_Rvs	CTCAACCCGGCCTTCGCTGGTCACCAGC
*cpxRA* complementation	cpxRA_orf_AgeI-Rvs	ATCAGCGTTATTAAGCACCGGTTTACGCGCGCTTATACAACGG
	cpxRA_orf_PmeI-Fwd	CGTTGCATGCCTGCAGTTTAAACCACTTGCTCCCAAAATCTTTCATG
mVenus cloning	mVen-pTrCmUltra-AgeI-Fwd	CCTATAATAGATTCATCACGTGTGAAAGAGGAGAAATCTAGAATGGTGAGCAAGGGCGAG
	mVen-pTrCmUltra-AgeI-Rvs	ACTATCAGCGTTATTAAGCACCGGTTACTTGTAGAGCTCGTCCATG
pECL-A/B screening	Eclo_pECLA_screen_Fwd	ATCCCCCTGACTGACATCCA
	Eclo_pECLA_screen_Rvs	CCCATATCTACCGTTCCCGC
	Eclo_pECLB_screen_Fwd	AAGCTTGCCTGAGAATGGCT
	Eclo_pECLB_screen_Rvs	TACTTCGGGAGAGGGATGGG
qRT-PCR (ATCC13047)	rpoD-qrtPCR-Fwd	ACTGCGTATGCGTTTCGGTA
	rpoD-qrtPCR-Rvs	AGCTACGCAGCACTTCAGAG
	degP-qrtPCR-Fwd	CAAGGACCCACGCTCTGATA
	degP-qrtPCR-Rvs	GCTGAAACAATCCCCGAGGT
	yebE-qrtPCR-Fwd	TTCTGTCCTGCTCTTCCACAC
	yebE-qrtPCR-Rvs	AGTACGAACCGCACATAGCG
	13047-rho-qRT-F	TTTTGGAGCCGGTATCGATAAGA
	13047-rho-qRT-R	GCCTACAACACCGTGGTTCC
qRT-PCR (AS02)	AS02-rho-qRT-F	GGTTGCGGAAATGGTCATCG
	AS02-rho-qRT-R	GTCGCGATAATGGTCAGGCT
	AS02-degP-qRT-F	ACCATTAAGGTGCAGATGAGCG
	AS02-degP-qRT-R	CGGTTTCGCCCAGACCAAAT
	AS02-yebE-qRT-F	CAATATGCCCGTCGCTCTTG
	AS02-yebE-qRT-R	TCGCGGAAACTGCTTACCAAA
qRT-PCR (AS06)	AS06-rho-qRT-F	TCGTCCATTTTGGAACCCGT
	AS06-rho-qRT-R	ACGTGATCATCCTGCTCGAC
	AS06-degP-qRT-F	TCGGGATCGGCTTTGCTATC
	AS06-degP-qRT-R	CCCAGAATCCCCAGTTCACC
	AS06-degP-yebE-F	CTGCTCACCAAAGTGGGGTT
	AS06-degP-yebE-R	TGGGACAGAAAACGTCGTCAT

### Generation of *E. cloacae cpxRA* knockout, *cpxA24*, and complemented strains

Scarless deletion of the *cpxRA* locus in *E. cloacae* ATCC 13047 used a two-step process relying on the pTOX6 plasmid system (72). Approximately 500 bp flanking regions were cloned to allow homologous recombination and deletion of most of the open reading frame of CpxRA. The KOD hot-start polymerase was used to generate flanking fragments, which were cloned in a three-way SLIC, into pTOX6 using *E. coli* MFDpir as the host with all steps including glucose (2 % wt/vol) and diaminopimelic acid (0.3 mM). Single crossovers were obtained by conjugation into *E. cloacae* with 3:1 ratio of overnight cultures of donor *E. coli* and recipient *E. cloacae* plated in a 100 µL spot on LB agar for 4 h at 37 °C, followed by collecting colonies, washing in 1 mL PBS, and plating serial dilutions on LB agar containing glucose (2 % wt/vol) and chloramphenicol (20 mg.L^−1^). Exconjugants were validated by PCR and inoculated in 5 mL M9 media + 2 % (wt/vol) rhamnose for 1 h at 37 °C to induce toxin expression, followed by plating serial dilutions on M9 agar plates supplemented with 2 % rhamnose and incubating at 37 °C overnight. Successful double crossovers were confirmed by patching resulting colonies on LB and LB + chloramphenicol (20 mg.L^−1^) plates. Chloramphenicol-sensitive colonies were screened by PCR and subjected to Sanger sequencing to confirm deletion of target genes.

The *cpxA24* strain containing a stable in-frame deletion of *cpxA* amino acids 93–124 in the type strain ATCC 13047 was generated as above using the pTOX6 allelic exchange system (72). Regions of approximately 500 bp flanking the chromosomal deletion site (containing the *cpxA24* allele) were amplified using KOD and inserted into pTOX6 at the XhoI site. Conjugation and crossover selection was carried out as described above.

To complement the *cpxRA* deletion strain, *cpxRA* and their natural promoter were cloned and integrated at a single copy at the Tn7 site downstream of *glmS*. The CpxRA and the upstream intergenic region containing the promoter were cloned into the PmeI-AgeI sites of pTraCam-EcloGlmS plasmid and conjugated into Δ*cpxRA E. cloacae* as above. CamR exconjugants were verified by PCR. A Δ*cpxRA E. cloacae* strain expressing mVenus from the same locus was used as a negative control. The presence of the endogenous pECL_A and pECL_B plasmids was confirmed in all strains used for experiments.

### Bacterial growth curves

Overnight cultures of the *E. cloacae* wild type, Δ*cpxRA,* Δ*cpxRA* + CpxRA, Δ*cpxRA* + Ven, or *E. cloacae cpxA24* were diluted to OD_600_ = 0.05 into 25 mL LB plus the appropriate antibiotics. Cultures were incubated at 37°C with shaking, and OD_600_ readings were taken hourly for 8 h.

### qRT-PCR

RNA was purified from ~1 × 10^9^ bacterial cells harvested during exponential phase (OD_600_ = 0.5) or stationary phase (OD_600_ = >3.0) where appropriate following the relevant treatments. When growing *E. cloacae* strains in 4 mM CuSO_4_, overnight cultures in LB were diluted 1:100 into fresh MHB + 4 mM CuSO_4_ and grown at 37 °C with shaking for around 3 h until the OD_600_ reached 0.5. The required volume of bacterial culture was directly added to 2× volume of RNAprotect Bacteria Reagent (Qiagen), mixed, and incubated at room temperature for 5 minutes before harvesting via centrifugation. All stabilized bacterial pellets were stored at −80 °C until use. RNA was purified using the PureLink RNA Mini Kit (Invitrogen) as per the manufacturer’s protocol. Following quantification of the RNA and assessment of purity using A260/A280 and A260/A230 ratios, 100 ng total RNA was taken for first-strand reverse transcription using the High-Capacity cDNA Reverse Transcription Kit (Applied Biosystems). The qRT-PCR reaction was carried out using SSO Advance SYBR Green Supermix (BioRad), using 0.2 µM primers (Table 4). qRT-PCRs were set up with at least two technical replicates and non-template controls. The thermal cycling conditions consisted of 40 cycles of 10 s at 95 °C followed by 60 s at 60 °C. The melting curves of each PCR product were analyzed to assess primer specificity. Relative expression levels of *degP* and *yebE* mRNAs were calculated using the ΔC_T_ method, using either *rpoD* or *rho* mRNA as a reference. All conditions were carried out in at least biological triplicate.

### RNA purification and sequencing

RNA was purified from exponentially growing cultures (OD_600_ = 0.5) of *E. cloacae* in LB alongside those of SCV grown to a similar OD_600_ and then left untreated or treated with 8 mg.L^−1^ gentamicin for 2 h. Experiments were performed on three separate occasions to collect biologically independent samples and purified together using the Qiagen RNeasy kit following manufacturer’s protocol. RNA sequencing was performed at the Advanced Sequencing facility at the Francis Crick Institute. Samples were normalized to 500 ng and ribosomal RNA was depleted with RiboMinus Bacteria 2.0 Transcriptome Isolation Kit (Invitrogen, Cat no. A47335), followed by library preparation with KAPA RNA HyperPrep kit (Roche, kit code KK8541), according to manufacturer instructions. The adapter concentration was 7 µM and the libraries were amplified by nine cycles of PCR. The quality and fragment size distributions of the purified libraries was assessed by a 4200 TapeStation Instrument (Agilent Technologies). Libraries were pooled and sequenced on the Illumina NovaSeq 6000 in single-read 100 configuration to a read depth of at least 22 M.

### Identification of differentially expressed genes and pathway enrichment

Sequencing analyses followed established pipelines (75). Quality control was performed using fastqc (76) and read filtering with fastp (77). Briefly, reads were mapped to the *E. cloacae* ATCC 13047 reference genome (GCA_000025565, genome assembly ASM2556v1 from Ensembl using the GTF annotation file ASM2556v1.49c) using kallisto (v.0.46.2) in conda run on a Windows PC with a Linux subsystem (78), followed by tximport (v.1.26.1) (79) to generate length scaled TPM counts mapped to genes in *E. cloacae* in R/Bioconductor (v.4.0 or higher). Genes with counts >1 in all nine samples were retained and normalized prior to obtaining log_2_ cpm in edgeR (v.3.40.1) (80). The study design included three conditions (parental, SCV, and SCV + gentamicin) and three repeats (nine total samples) with experiment as a blocking factor using “duplicateCorrelation” function in limma. Differentially expressed genes (SCV vs parental, and SCV + gentamicin vs SCV contrasts) with false discovery rate (FDR)-adjusted *P*-value ≤0.01 with log_2_ fold-change ≥2 or ≤ −2 were generated with topTable in limma (v.3.54.0) (81).

gProfiler [online and R; (82)] was used for the enrichment of pathways using GMT files obtained from Ensembl or a manually curated GMT file containing lists of transcription factors and their target genes (33). Transcription factors and their targets from the curated *E. coli* MG1655 genome in BioCyc (83) were mapped to the genome of *E. cloacae* ATCC 13047, and converted into a GMT file to use with gProfiler, including newly identified CpxRA targets in *E. coli* (33). Wrangling of these tables was performed using power queries in Excel. GO terms and transcription factors with FDR-adjusted *P*-value ≤0.05 are shown in figures (84).

### Data collection and statistical analysis

For all experiments, a single mean was calculated from technical replicates (e.g., duplicate dilutions for MICs or serial dilutions for enumeration of viable CFU). Experiments were repeated independently on different days to obtain statistically independent means for different groups that were then compared in R (v.4.0 or higher) or GraphPad Prism (version 8.0 or higher). In the uncommon case of obtaining different MICs between technical duplicates, the highest concentration of the two was noted. Due to the unstable and heterogenous nature of heteroresistance, each colony of SCV and non-SCV on an agar plate was considered biologically independent, and typically two to three colonies were tested on a given day. Experiments were repeated independently on different days with different batches of SCVs and non-SCVs throughout this study, and all data were pooled for statistical analyses.

Student’s two-tailed *t*-tests were used when comparing two groups. Non-parametric Mann-Whitney *U*-test was used when comparing MIC of two groups, followed by FDR-based correction of *P*-values for multiple comparisons (α = 0.05). Aligned-rank transformation tool (ARTool package in R) was used for factorial analysis of variance (ANOVA) of MIC data without missing values and when F values of ANOVAs on aligned responses not of interest were 0. For more than two groups, factorial ANOVA (linear mixed-effects models) was calculated with R packages lme4 (85) and emmeans (86) as implemented in the grafify package (87). Mixed-effects models used “Experiment” as blocking factor with random intercepts, and FDR adjustment (α = 0.05) was used to correct *P*-values for multiple comparisons. For survival assays, log transformations were typically required to ensure model residuals were approximately normally distributed following model diagnostics [e.g., using ggResidpanel (88)]. Mean ± SD or median ± interquartile range (IQR) (boxes indicating IQR, line at median, whiskers 1.5× IQR) are shown as indicated in figure legends. Data plotting used grafify and ggplot2 packages in R (89).

## Data Availability

The raw mRNA sequencing data are in BioProject PRJNA988129 and processed data under accession GSE236124 in the GEO database. Request for strains or plasmids generated in this study should be made to and will be fulfilled by ARS (a.shenoy@imperial.ac.uk).

## SUPPLEMENTAL MATERIAL

Supplemental Figures mbio.01699-24-s0001.pdf Figures S1 to S3.

Supplemental Tables mbio.01699-24-s0002.xlsx Tables S1 to S3, related to RNA sequencing data.

ASM does not own the copyrights to Supplemental Material that may be linked to, or accessed through, an article. The authors have granted ASM a non-exclusive, world-wide license to publish the Supplemental Material files. Please contact the corresponding author directly for reuse.
