# Supplementary material for: Aminoglycoside heteroresistance in Enterobacter cloacae is driven by the cell envelope stress response
Source: mBio. 2024 Oct 30;15(12):e01699-24. doi: 10.1128/mbio.01699-24 (PMC11633387; doi:10.1128/mbio.01699-24)
Supplement: Supplemental Figures — Figures S1 to S3. [file mbio.01699-24-s0001.pdf]

Supplementary Figures

Aminoglycoside heteroresistance in *Enterobacter cloacae* is driven by the cell envelope stress response.

Ana J. Choi[1,2 $], Daniel J. Bennison[1,2 $], Esha Kulkarni[1,2], Hibah Azar[1,2], Haoyu Sun[1,2], Hanqi Li[1,2], Jonathan Bradshaw[1,2], Hui Wen Yeap[1,2], Nicholas Lim[1,2], Vishwas Mishra[1,2], Anna Crespo-Puig[1,2], Ewurabena A. Mills[3], Frances Davies[3], Shiranee Sriskandan[1, 2, 3], Avinash R. Shenoy[1, 2, 4, *]

[1] Department of Infectious Disease, [2] Centre for Bacterial Resistance Biology, Imperial College London, London, UK, [3] NIHR Health Protection Research Unit in Healthcare Associated Infections and Antimicrobial Resistance, Imperial College London, London, UK, [4] The Francis Crick Institute, London, UK

[$] equal contribution

* Correspondence & Lead Contact:

Address: Room 4.40A, Flowers Bldg, Armstrong Road, MRC CMBI, Imperial College London, London SW7 2AZ, UK

Email: a.shenoy@imperial.ac.uk

Figure S1

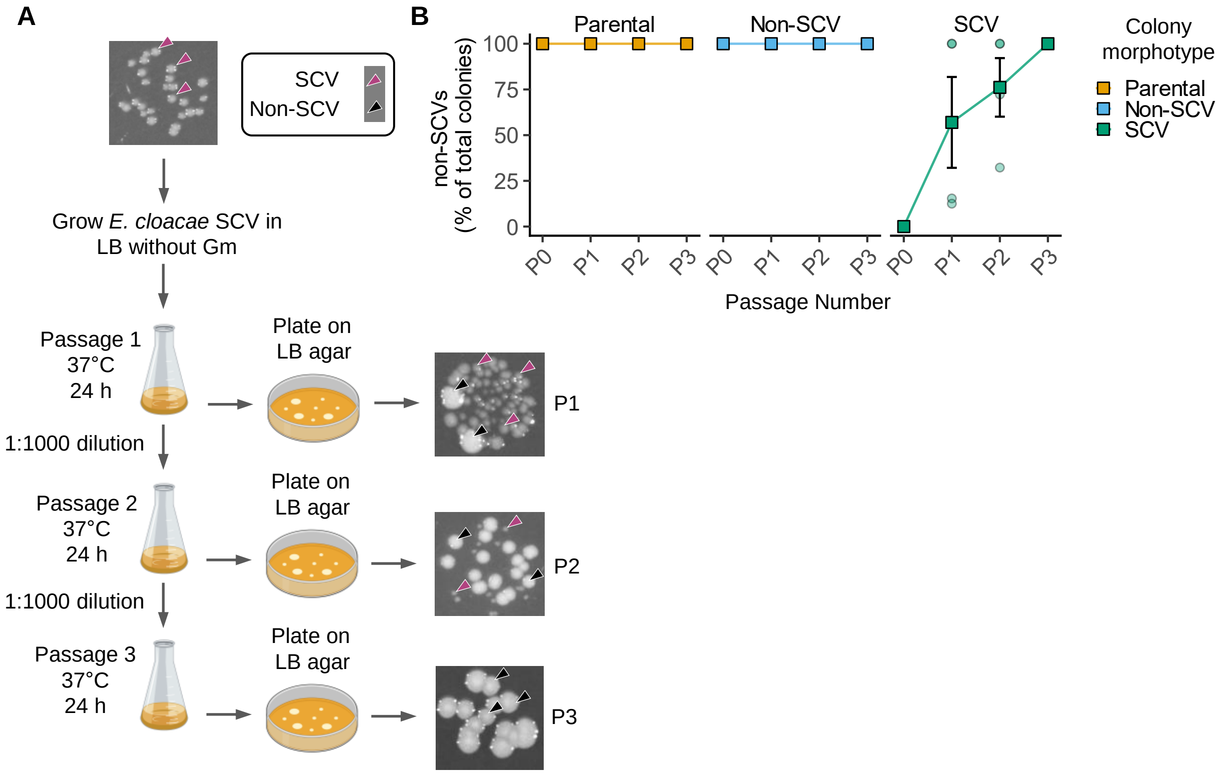

Figure S1. SCVs fully revert after passages in antibiotic-free medium. (A) Schematic showing the outline of experiments to assess the time taken for the reversion of SCVs into non-SCVs following growth in antibiotic-free LB broth. As described in ***Figure 2A***, SCV and non-SCV colonies were initially obtained by treating *E. cloacae* with 20 $mg.L^{-1}$ gentamicin for 24 h followed by plating on LB agar without antibiotics. Four representative colonies of SCV, non-SCV and 'parental' (colonies from culture not exposed to gentamicin) morphotypes were inoculated into LB flasks and grown overnight. Each flask was passaged at 1:1000 dilution at 24 h intervals, followed by plating on LB agar (20 µl drops of serial 10-fold dilutions). Schematic in **A** depicts experiment with SCVs, and representative images at each passage show reversion to non-SCVs. Arrows used to depict colony morphotypes indicated in the legend. Images are representative of four biological replicates. **(B)** Quantification of experiments in **A**, showing relative frequency of non-SCVs at every passage. P1, P2 and P3 refer to Passage 1, 2 and 3, respectively. Square boxes represent the mean value of n = 4 biological replicates (each replicate shown as a circle), and error bars represent SD.

Figure S2

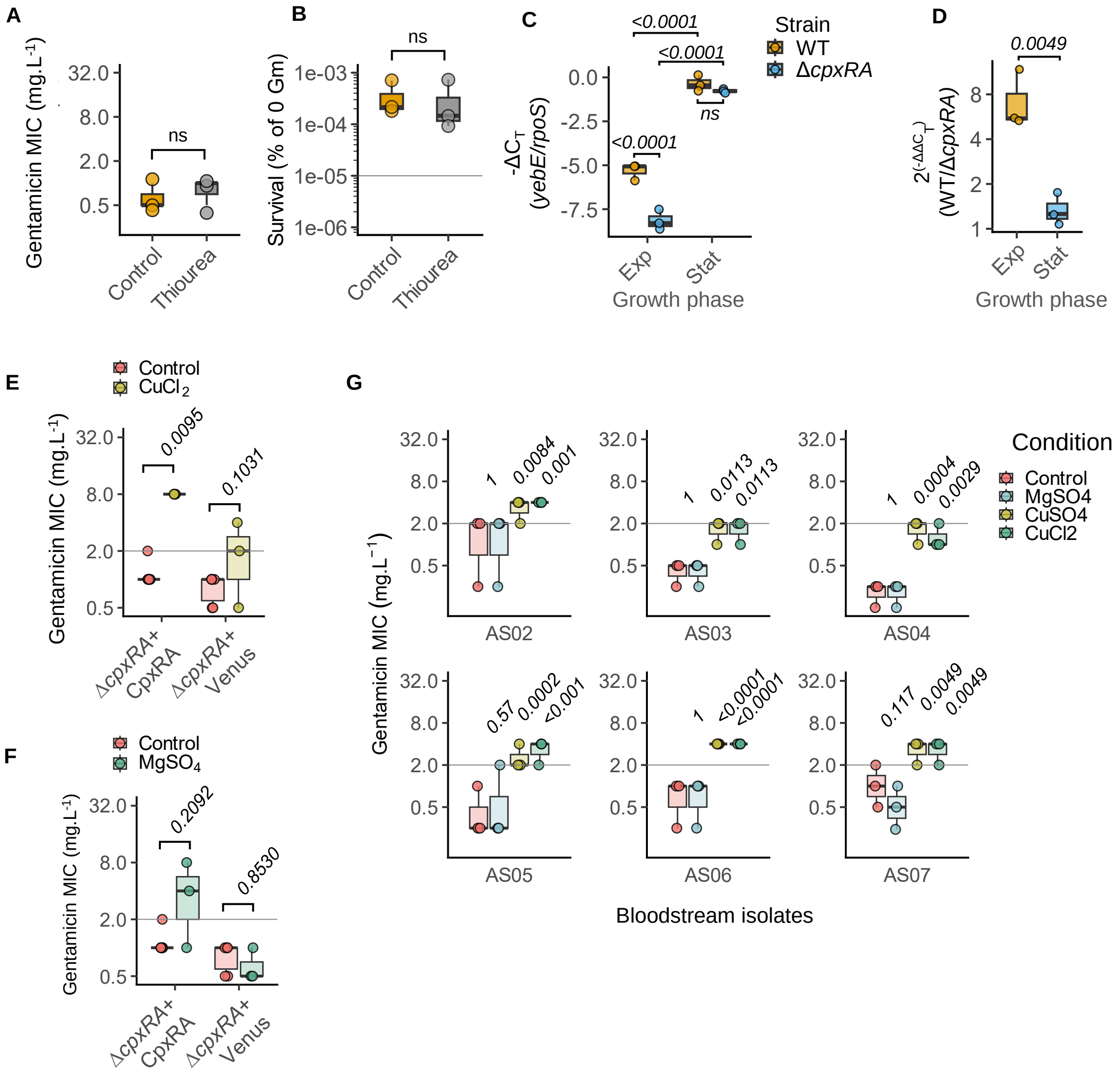

Figure S2. Effect of thiourea and metals on the ability of *E. cloacae* to overcome gentamicin. (A-B) Gentamicin MIC (**A**) and population analysis profiling (PAP; **B**) of *E. cloacae* in the absence and presence of thiourea (75 mM). Data from n = 3 independent experiments. ns, not significant ($P > 0.05$). **(C-D)** qRT-PCR showing the expression of the CpxRA target gene *yebE* in wild type and Δ*cpxRA* *E. cloacae* during exponential phase (Exp; OD_{600} = 0.5) or stationary phase (Stat; OD_{600} = 3.0) growth, as labelled. Expression of *yebE* relative to *rho* ($-\Delta C_T$) is shown in **C**, and relative fold-change ($2^{-\Delta\Delta CT}$) in wild-type bacteria compared to Δ*cpxRA* is shown in **D**. Data from n = 3 independent experiments. Two-tailed *P* values from mixed-effects ANOVAs. **(E-F)** Gentamicin MIC for the complemented and Δ*cpxRA* knockout strains as indicated in the absence or presence of copper chloride (**E**) or magnesium sulphate (**F**; 4 mM each). Data from n = 3 independent experiments. Two-tailed *P* values for the indicated comparisons from mixed effects ANOVAs. **(G)** Gentamicin MIC for the bloodstream isolates AS02-AS07 in the absence (Control) or presence of the indicated copper or magnesium salts (4 mM). Data from n = 3 independent experiments. Two-tailed *P* values for the indicated comparisons from non-parametric ANOVA following aligned-rank transformation. In **A-G**, horizontal line is the median, box shows the IQR and whiskers depict 1.5xIQR.

Figure S3

```
                                    Transmembrane domain          Periplasmic region
E.clo ATCC13047    MIGSLTARIFAIFWLTLALVLMLVLMLPKLDSRQMTELLDSEQRQGVMIEQHVEAELAND   60
K.pne NCTC9633     MIGSLTARIFAIFWLTLALVLMLVLMLPKLDSRQMTELLESEQRQGIMIEQHVEAELAND   60
E.col MG1655      MIGSLTARIFAIFWLTLALVLMLVLMLPKLDSRQMTELLDSEQRQGLMIEQHVEAELAND   60
STm SL1344       MIGSLTARIFAIFWLTLALVLMLVLMLPKLDSRQMTELLDSEQRQGLMIEQHVEAELAND   60
                 ***************************************:******:*************
                                                        cpxA24 deletion (Δ93-124)
E.clo ATCC13047    PPNDLMWWRRLFRAIDKWAPPGQRLLLVTSEGRVIGADRNEMQIIRNFIGQADNADHPQK   120
K.pne NCTC9633     PPNDLMWWRRLFRAIDKWAPPGQRLLLVTSEGRVIGAERNEMQIIRNFIGQADNADHPQK   120
E.col MG1655      PPNDLMWWRRLFRAIDKWAPPGQRLLLVTTEGRVIGAERSEMQIIRNFIGQADNADHPQK   120
STm SL1344       PPNDLMWWRRLFRAIDKWAPPGQRLLLVTSEGRVIGAERSEMQIIRNFIGQADNADHPQK   120
                 *****************************:*******:*.********************
                                                             Transmembrane domain
E.clo ATCC13047    KKYGRVEMVGPFSVRDGEDNYQLYLIRPASSSQSDFINLLFDRPLLLLIVTMLVSSPLLL   180
K.pne NCTC9633     KRYGRLEMVGPFSVRDGEDNYQLYLIRPASTSQSDFINLLFDRPLLLLIVTMLVSAPLLL   180
E.col MG1655      KKYGRVELVGPFSVRDGEDNYQLYLIRPASSSQSDFINLLFDRPLLLLIVTMLVSTPLLL   180
STm SL1344       KKYGRVEMVGPFSVRDGEDNYQLYLIRPASSSQSDFINLLFDRPLLLLIVTMLVSSPLLL   180
                 *:***:*:**********************:************************:****

E.clo ATCC13047    WLAWSLAKPARKLKNAADEVAQGNLRQHPELESGPQEFLAAGTSFNQMVSALDRMMTAQQ   240
K.pne NCTC9633     WLAWSLAKPARKLKNAADEVAQGNLRQHPELEAGPQEFLAAGASFNQMVTALERMMTSQQ   240
E.col MG1655      WLAWSLAKPARKLKNAADEVAQGNLRQHPELEAGPQEFLAAGASFNQMVTALERMMTSQQ   240
STm SL1344       WLAWSLAKPARKLKNAADEVAQGNLRQHPELEAGPQEFLAAGASFNQMVTALERMMTSQQ   240
                 ********************************:*********:******:**:****:**

E.clo ATCC13047    RLLSDISHELRTPLTRLQLGTALLLRRSGESKELERIETEAHRLDSMINDLLVMSRNQQK   300
K.pne NCTC9633     RLLSDISHELRTPLTRLQLGTALLRRRSGESKELERIETEAHRLDSMINDLLVMSRNQAK   300
E.col MG1655      RLLSDISHELRTPLTRLQLGTALLRRRSGESKELERIETEAQRLDSMINDLLVMSRNQQK   300
STm SL1344       RLLSDISHELRTPLTRLQLGTALLRRRGGESKELERIETEAQRLDSMINDLLVMSRNQQK   300
                 ************************ **.*************:**************** *

E.clo ATCC13047    NALVSETVKANHLWHEVLDNAAFEAEQMGKSFTVNFPPGPWPLYGNPNTLESALENIVRN   360
K.pne NCTC9633     NALVSETVKANQLWNEVLDNAAFEAEQMGKSFTVEYPPGPWPLYGNPNALESALENIVRN   360
E.col MG1655      NALVSETIKANQLWSEVLDNAAFEAEQMGKSLTVNFPPGPWPLYGNPNALESALENIVRN   360
STm SL1344       NALVSETMKANQLWGEVLDNAAFEAEQMGKSLTVNYPPGPWPLYGNPNALESALENIVRN   360
                 *******:***:** ****************:**::************:***********

E.clo ATCC13047    ALRYSHTKIEVAFSVDKDGITVIVDDDGPGVSPEDREQIFRPFYRTDEARDRESGGTGLG   420
K.pne NCTC9633     ALRYSHTKISVSFSVDKDGITVNVDDDGPGVSPEDREQIFRPFYRTDEARDRESGGTGLG   420
E.col MG1655      ALRYSHTKIEVGFAVDKDGITITVDDDGPGVSPEDREQIFRPFYRTDEARDRESGGTGLG   420
STm SL1344       ALRYSHTKIKVGFSVDKDGITITVDDDGPGVSPEDREQIFRPFYRTDEARDRESGGTGLG   420
                 *********.*.*:*******: ************************************

E.clo ATCC13047    LAIVETAMQQHRGWVKADDSPLGGLRLTLWLPLYKRS   457
K.pne NCTC9633     LAIVETAIQQHRGWVKADDSPLGGLRLTIWLPLYKRT   457
E.col MG1655      LAIVETAIQQHRGWVKAEDSPLGGLRLVIWLPLYKRS   457
STm SL1344       LAIVESAMQQHRGWVKADDSPLGGLRLTLWLPLYKRT   457
                 *****:*:*********:*********.:*******:
```

Figure S3. Sequence alignments of representative CpxA proteins. Amino acid sequence alignments of the CpxA proteins from *E. cloacae* ATCC13047 (*E.clo*), *K. pneumoniae* NCTC9633 (*K.pne*), *E. coli* MG1655 (*E.col*) and *Salmonella* Typhimurium SL1344 (*S*Tm). The *cpxA24* deletion site (aa 93-124) is highlighted (purple), along with the periplasmic region (blue) and transmembrane domains (underlined). The multiple sequence alignment was generated using Clustal Omega.
